# Physiological and transcriptomic analyses reveal a response mechanism to cold stress in *Santalum album* L. leaves

**DOI:** 10.1038/srep42165

**Published:** 2017-02-07

**Authors:** Xinhua Zhang, Jaime A. Teixeira da Silva, Meiyun Niu, Mingzhi Li, Chunmei He, Jinhui Zhao, Songjun Zeng, Jun Duan, Guohua Ma

**Affiliations:** 1 Key Laboratory of Plant Resources Conservation and Sustainable Utilization, South China Botanical Garden, Chinese Academy of Sciences, Guangzhou, China; 2 Independent Researcher, P. O. Box 7, Miki cho post office, Ikenobe 3011-2, Miki-cho Kagawa-Ken, 761-0799, Japan; 3 Genepioneer Biotechnologies Co. Ltd., Nanjing 210014, China

## Abstract

*Santalum album* L. (Indian sandalwood) is an economically important plant species because of its ability to produce highly valued perfume oils. Little is known about the mechanisms by which *S. album* adapts to low temperatures. In this study, we obtained 100,445,724 raw reads by paired-end sequencing from *S. album* leaves. Physiological and transcriptomic changes in sandalwood seedlings exposed to 4 °C for 0–48 h were characterized. Cold stress induced the accumulation of malondialdehyde, proline and soluble carbohydrates, and increased the levels of antioxidants. A total of 4,424 differentially expressed genes were responsive to cold, including 3,075 cold-induced and 1,349 cold-repressed genes. When cold stress was prolonged, there was an increase in the expression of cold-responsive genes coding for transporters, responses to stimuli and stress, regulation of defense response, as well as genes related to signal transduction of all phytohormones. Candidate genes in the terpenoid biosynthetic pathway were identified, eight of which were significantly involved in the cold stress response. Gene expression analyses using qRT-PCR showed a peak in the accumulation of *SaCBF2* to *4*, 50-fold more than control leaves and roots following 12 h and 24 h of cold stress, respectively. The CBF-dependent pathway may play a crucial role in increasing cold tolerance.

Low temperature is one of the major abiotic factors that impedes plant growth and limits crop yield and geographical distribution^1^. Plants have evolved several adaptive mechanisms to cope with cold stress to protect them against damage^2^. A diverse range of plant species can withstand cold stress to varying degrees, but this depends on reprogramming gene expression to adjust their metabolism and developmental programs^3^. Not all plants are able to tolerate low nonfreezing temperatures while temperate plants can adapt to a cold environment and increase their cold tolerance in response to low temperatures, such as Arabidopsis, winter wheat (*Triticum aestivum* L.) and barley (*Hordeum vulgare* L.), among others^4^. By contrast, plants of tropical and subtropical origin generally have little tolerance to low temperatures and exhibit various symptoms of chilling injury, such as maize (*Zea mays* L.)^5^, cassava (*Manihot esculenta* Crantz)^6^, and soybean (*Glycine max* Merr.)^7^.

The mechanism of cold stress in plants has been extensively studied over the last 20 years but the complete mechanisms by which plants perceive low temperatures still remain unknown. Research has shown that cold stress tolerance involves the remodeling of cell structures and reprogramming gene expression by triggering a series of protective mechanisms against cold damage, including an increase in the level of intracellular solutes, the accumulation of cryoprotectants and antioxidants, as well as the induction of antifreeze proteins and cold-regulated (COR) proteins^8^,^9^. During cold stress, these metabolites and proteins help to protect plant membranes and prevent cell disruption by stabilizing membrane lipids, maintaining ion homeostasis and scavenging reactive oxygen species (ROS)^3^.

The molecular mechanisms underlying cold stress tolerance have been investigated in the model plant Arabidopsis, as well as in other crop species such as maize, wheat and barley^1^,^10^,^11^. Plants have developed C-repeat (CRT)-binding factor/dehydration-responsive element binding factor (CBF/DREB)-dependent and CBF/DREB-independent pathways, allowing them to adapt to cold stress. The most extensively studied mechanism of the response to cold has focused on the CBF/DREB-mediated transcriptional regulatory cascade^4^. Recently, it was revealed that the number of genes (about 100) induced by *CBF* genes only accounted for a small percentage of *COR* genes in freezing tolerance while other cold-induced genes showed expression patterns similar to those of *CBF*s in Arabidopsis, such as *ZAT12 (C2H2*-*type zinc finger*), *RAV1 (related to ABI3*/*VP1*), and *MYB73*^12^. Besides Arabidopsis, the CBF/DREB1 transcription factors (TFs) are present in a wide array of plants, including crops, such as wild tomato (*Solanum habrochaites* S. Knapp & D.M. Spooner) and rice (*Oryza sativa* L.)^13^,^14^, as well as woody plants, such as grey poplar (*Populus tremula* × *alba*)^15^, frost grape (*Vitis riparia* Michx.)^16^, silver birch (*Betula pendula* Roth.)^17^, and cider gum (*Eucalyptus gunnii* Hook.f.)^18^. In addition, signaling molecules such as Ca^2+^, protein kinase, ROS, and hormones (auxin, abscisic acid (ABA), and gibberellin (GA)) play important roles in cold-induced gene expression^19^,^20^.

*Santalum album* L., commonly known as Indian sandalwood, is the best known economically important member of the Santalaceae, and is highly valued for its aromatic heartwood that contains sandal oil, which has various applications in cosmetics, religion and medicine^21^,^22^. Four sesquiterpenols (α-, β-, and *epi*-β-santalol, and α-*exo*-bergamotol) make up approximately 90% of *S. album* sandalwood oil^21^. Relevant terpenoid synthase genes producing these major constituents were cloned and characterized^23^,^24^,^25^,^26^,^27^,^28^. Külheim *et al*. discovered that several key genes in the terpenoid pathway were induced by spraying a 0.1% methyl jasmonate solution over the foliage, but valuable heartwood essential oils were not be detected in the leaves and stems of seedlings following this treatment^29^. Whether environmental stresses, including cold stress, affect the expression of terpene synthase genes and the accumulation of essential oil in sandalwood is unclear.

*S. album* is a tropical and subtropical species, and cold damage is the main factor limiting the introduction and cultivation of this species. It occurs in coastal dry forests and also grows at 700 m above sea level in India and displays a wide temperature tolerance ranging from 4.5 to 38 °C^30^. As the price for the heartwood of *S. album* has increased at an average rate of more than 50% per year over the past two decades^31^, natural populations have been heavily exploited and thus wild populations are vulnerable to extinction^32^. *S. album* is now cultivated in South China, Sri Lanka, Indonesia, Malaysia, the Philippines and Northern Australia^33^. This species was first introduced to South China Botanical Garden (SCBG) in 1970 and its cultivation was then limited to Guangdong, Fujian, and Guangxi provinces, which have a subtropical climate. Every year, these regions experience extremely low temperatures (<−2 °C) in January. A statistical survey showed that these trees grow safely above −1 °C throughout winter^34^. However, little is known about the physiological and molecular mechanisms that underlie the cold stress response in *S. album*.

In this study, we completed the in-depth transcriptome sequencing of leaf tissues of *S. album* seedlings. Comprehensive and dynamic changes in the leaves in response to cold stress were monitored using physiological and RNA-seq analyses. Genes involved in terpenoid biosynthesis were identified from the transcriptome data. Eight genes were differentially expressed by cold stress. We further investigated the TFs involved in regulating the response of sandalwood to cold stress using transcriptome data combined with RACE and quantitative real time PCR (qRT-PCR). This study presents the first global transcriptomic overview of the genes involved in the cold stress response in sandalwood. Our findings lay a solid foundation for further investigation of the molecular mechanisms responsible for this tree’s response to cold stress.

## Results

### Morphological and physiochemical changes of *S. album* in response to cold stress

**Figure 1:**
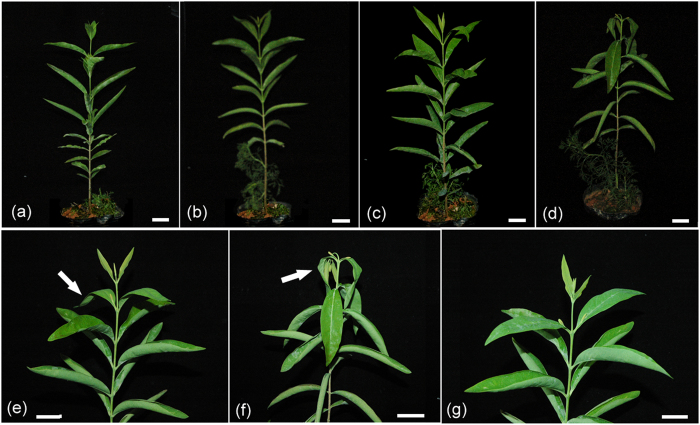
Phenotypic responses of six-month-old sandalwood seedlings after 4 °C treatment. (**a**) Control plant. (**b**–**d**) Cold-stressed plants for 12, 24, and 48 h, respectively. (**e**,**f**) The terminal shoots of seedlings at 24 h (**e**) and 48 h (**f**) after cold treatment. Arrows show leaves harmed by cold. (**g**) Seedlings recovered within 48 h after cold treatment. Potted host plant in (**a,b,c,d**) is *Kuhnia rosmarnifolia*. Scale bar = 2 cm.

**Figure 2:**
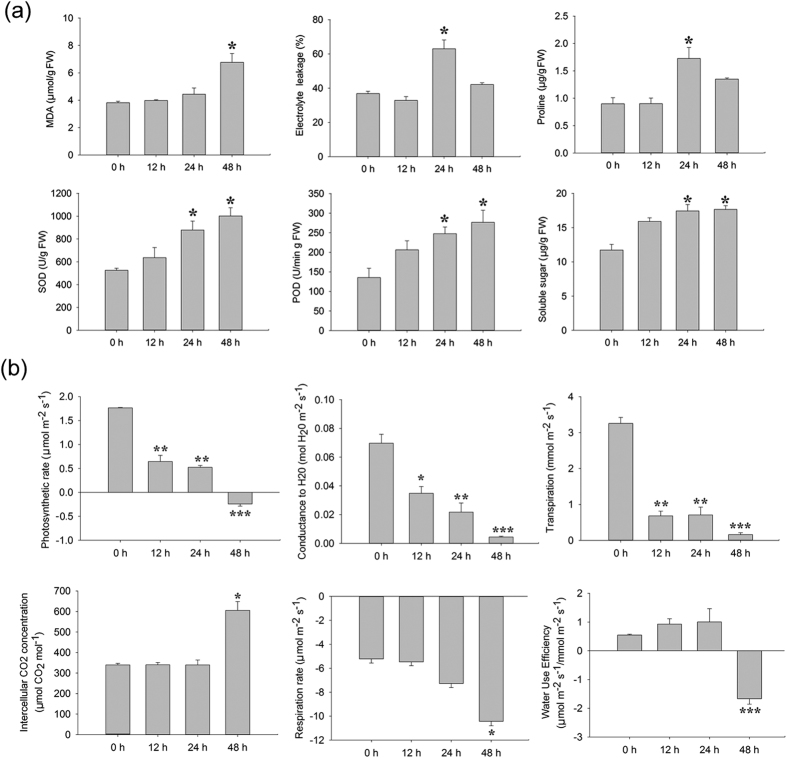
Physiological and phytochemical responses of *Santalum album* leaves exposed to 4 °C. (**a**) Changes in malondialdehyde (MDA), electrolyte leakage, proline content, superoxide dismutase (SOD) activity, peroxidase (POD) activity, and soluble sugar content in response to cold stress; (**b**) Photosynthetic indices under cold stress. Three measurements were averaged and statistically treated using a *t*-test. *P ≤ 0.05 > 0.005; **P ≤ 0.005 < 0.001; ***P < 0.001. FW, fresh weight.

Sandalwood seedlings displayed no obvious morphological alterations prior to 24 h of cold stress (Fig. 1a,b), but young leaves began to wilt as cold treatment was prolonged from 24 to 48 h (Fig. 1c–f). The terminal shoots of seedlings recovered within 48 h after being returned to normal conditions (Fig. 1g). Malondialdehyde (MDA) and electrolyte leakage (EL) are generally used as direct stress markers to reflect membrane damage by cold stress^35^. In this study, MDA content showed no significant change at 12 h (relative to the control) and a slight increase at 24 h after cold treatment, but peaked at 48 h with a 1.77-fold higher change than the control (Fig. 2a). EL decreased slightly at 12 h, increased dramatically at 24 h, then decreased after 48 h of cold stress. The content of proline, a well-known osmoprotectant, showed no significant difference at 12 h compared to the control, but increased rapidly and peaked at 24 h, then decreased slightly after 48 h of cold stress. These results strongly suggest that the cold-stressed sandalwood seedlings actively mounted a stress response after they were exposed to 4 °C. Moreover, the activities of superoxide dismutase (SOD) and peroxidase (POD) showed continuously increasing trends, with approximately two-fold higher levels than the control at 48 h, implying that these ROS-scavenging enzymes were involved in the detoxification of ROS induced by cold. The total soluble carbohydrate content increased at 12 h, but did not change significantly after 24 and 48 h of exposure to cold.

The dynamic net photosynthetic rate (A), H_2_O stomatal conductance (Gs), transpiration rate (E), intercellular CO_2_ concentration (Ci), and respiration rate (R) of leaves were further monitored during cold stress (Fig. 2b). The level of A decreased sharply at 12 h after exposure to cold and declined gradually from 12 to 24 h, but reached a minus value at 48 h. Both Gs and E showed a similar trend as A. A stable level of Ci was sustained from 0 to 24 h, but increased sharply by 60–80% at 48 h. It is likely that the rapid decline in A caused much CO_2_ to fill the intercellular spaces. Water-use efficiency (WUE) was higher at 12 and 24 h compared to the control, implying that an increase in WUE occurred in response to moderate water deficits caused by cold. However, WUE became sharply minus after 48 h of cold stress, indicating that plants suffered from severe cold stress. R decreased gradually from 0 to 48 h.

### Development of *de novo* transcriptome reference sequence and mapping of stress-induced transcriptome sequences from sandalwood leaves

In total, 97,349,766 clean reads out of 100,445,724 raw reads (GenBank Accession: SRR3731808, SRR3731809) were obtained from *S. album* leaves after adapter trimming and low quality trimming. *De novo* sequences were assembled to yield the *S. album* Gene Index Version 2 (SaGI02) with a total of 135,938 non-redundant unigenes, having an average size of 1203 bp and an N50 value of 2253 (Table S1). The length distributions of unigenes revealed that more than 60,507 unigenes (44.51%) were greater than 1000 bp (Fig. S1). A total of 80,426 sequences (59.16%) were annotated as homologs against the NCBI non-redundant protein (Nr) database, Swiss-Prot, Kyoto Encyclopedia of Genes and Genomes (KEGG), eukaryotic orthologous groups (COG) and GO databases with a cut-off E-value of <10^−5^. For the E-value distribution, more than half of the homologous sequences (50.5%) had a threshold E-value less than 1.0E^−60^ that showed strong homology (Fig. S2a). The similarity distributions showed that 68.9% of sequences had more than 60% similarity with sequences within the Nr database, while 31.1% of matches had lower similarity that ranged from 17% to 60% (Fig. S2b). The species that provided the best BLASTx match was *V. vinifera*, which showed 52.7% homology (Fig. S2c).

To gain a dynamic view of the gene profiles regulated by cold stress, we further performed RNA-seq analysis of non-cold-treated leaves (0 h) and cold-treated leaves at 12, 24, and 48 h using three biological replicates. A total of 12 libraries that were constructed were subjected to the Illumina sequencing platform. The sequenced raw reads have been submitted to the SRA at NCBI with the following accession numbers: SRR3490434, SRR3490436, SRR3490438 through to SRR3490442, SRR3490480, SRR3490561, SRR3490688, SRR3490711, and SRR3490712. After filtering out low-quality data (tags containing unknown base N and only adaptor tags), the clean reads of all 12 libraries accounted for more than 99.9% of raw reads (Fig. S3). Approximately 89.87–91.45% of reads in the 12 libraries were mapped to SaGI02, suggesting that the transcriptome was a reliable reference, in which approximately 72.24–74.14% of reads were perfectly matched (Table 1).

### Correlation analysis of samples and expression pattern of differentially expressed genes under cold treatment

**Figure 3:**
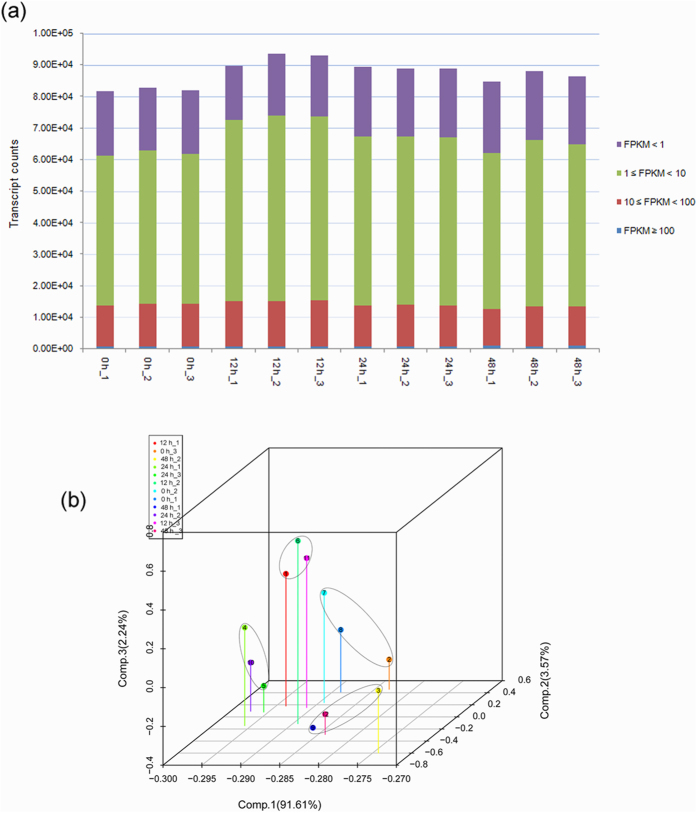
Global analysis of transcriptome datasets of biological replicates. (**a**) Bar plot describing the number of expressed transcripts after filtering. (**b**) Principal component analysis (PCA) of transcriptome data; each dot represents a sample with the principal component value.

**Figure 4:**
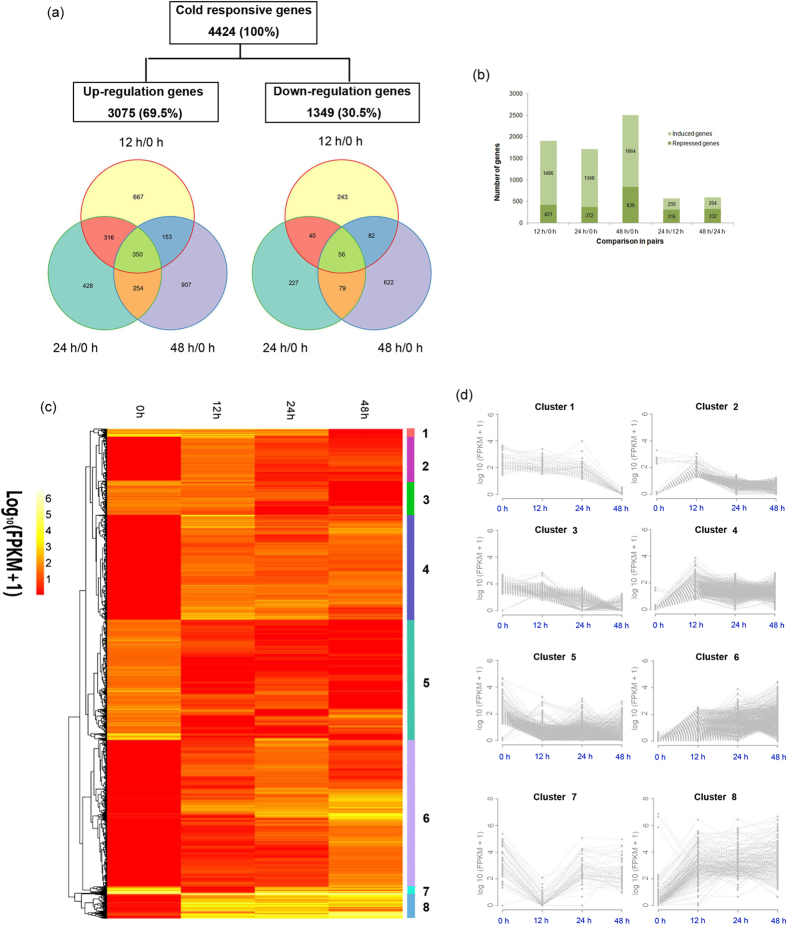
Expression profiling of cold (4 °C)-regulated differentially expressed genes (DEGs) in *S. album*. (**a**) Venn diagrams represent the overlaps of induced (left) and repressed (right) genes across three comparisons. (**b**) Induced and repressed genes for each pairwise comparison. (**c**) Heat map of the expression profiles of 4424 DEGs after cold stress. (**d**) Hierarchical clustering of DEGs in clusters in Fig. 3(c). The X axis represents time points of cold treatments. The Y axis represents the value of the relative expression level (log2 (FPKM + 1))(Fragments Per Kilobase of transcript per Million mapped reads). Hierarchical clustering was performed for DEGs with a ≥2-fold change and deviation probability ≥0.8 in at least one pairwise comparison of 0 vs 12 h/24 h/48 h using the Manhattan distance matrix of the heatmap.2 function in the gplots package of the R environment.

The global abundance of gene expression showed that most transcripts were activated by cold, in which the number of genes with FPKM, i.e. Fragments Per Kilobase of transcript per Million mapped reads, ranging from 1 to 10 was most abundant, as assessed by box plot analysis (Fig. 3a). The square of Pearson’s correlation coefficient (R^2^) was greater than 0.92 among the three biological replicates at each time point, indicating both operational stability and the reliability of the experimental results (Table S2). Principal component analysis showed that the expression levels between all biological replicates at each time point were highly correlated (Fig. 3b). These data suggest that alterations in quantitative expression could be estimated by statistical analysis of this dataset. A total of 4,424 differentially expressed genes (DEGs) were identified as being cold responsive over 48 h of cold stress, including 3,075 cold-inducible and 1,349 cold-repressed genes (Fig. 4a,b). A heat map representing the transcript levels for these transcripts at 0, 12, 24 and 48 h showed that these transcripts could be divided into eight clusters based on the modulation of their expression (Fig. 4c,d; Table S3). Most genes in cluster 2 were rapidly up-regulated at 12 h, but were down-regulated 24 and 48 h post cold treatment, indicating that these transcripts were transiently expressed by cold. Clusters 4, 6 and 8 showed similar patterns of changes in transcript, with an increase in expression at 12, 24, and 48 h compared to the control.

### Gene ontology and pathway enrichment analyses of differentially expressed genes

**Figure 5:**
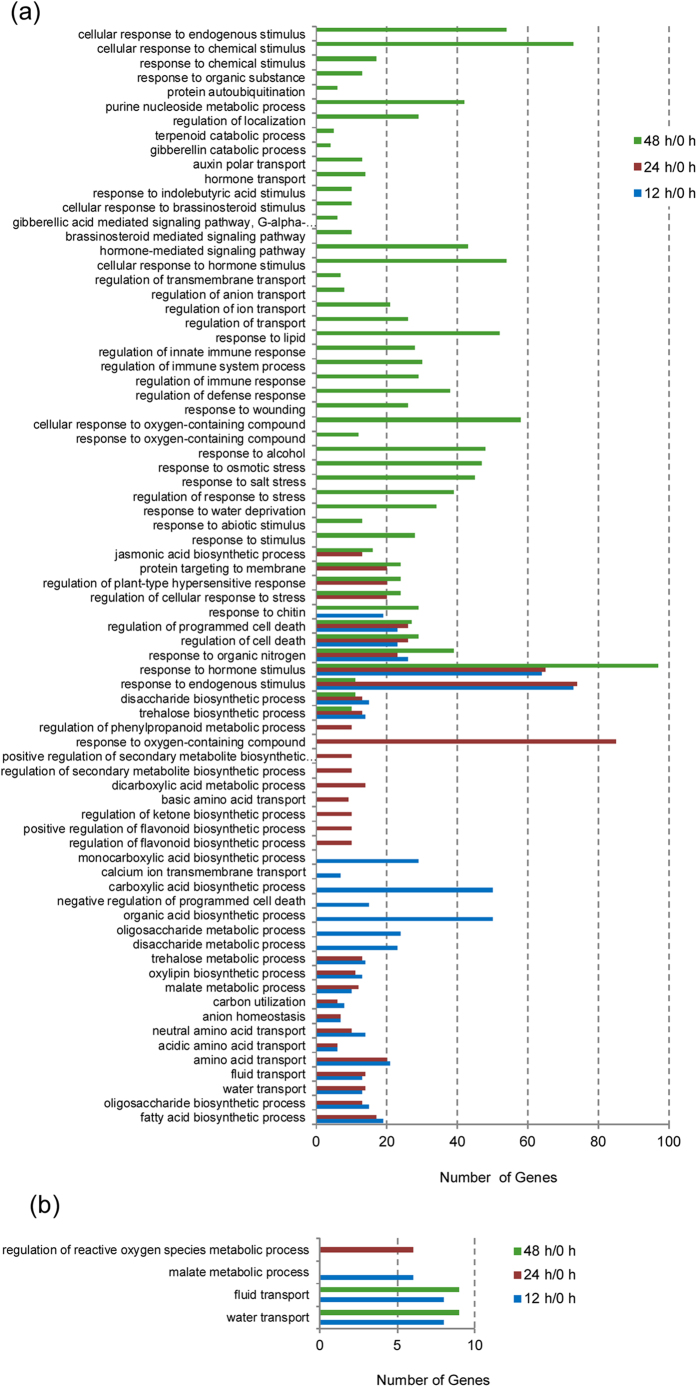
Gene ontology-enriched categories for biological processes among transcripts induced or repressed by cold (4 °C). (**a**) Processes enriched for cold-induced transcripts. (**b**) Processes enriched for cold-repressed transcripts. The induced or repressed DEGs were annotated to the GO database using a hypergeometric test. The calculated *P* value underwent a Bonferroni correction and the threshold of the corrected *P* value was less than 0.05.

In order to identify the biological functions associated with the cold stress response in sandalwood leaves, pairwise gene set enrichment analyses using a hypergeometric test were performed to search for over- and under-represented GO terms (0 vs 12 h/24 h/48 h). The datasets of three time points in comparison with the control yielded many significant over-represented GO terms (Fig. 5; Table S4). As shown in Fig. 5a, there were seven over-represented GO terms in all three pair-wise comparisons. Some overlapping and unique GO terms were observed at different time points, suggesting that a dynamic response occurred over the time course of cold stress. Prior to 24 h of cold stress, GO terms enriched in up-regulated transcripts were associated with plasma membrane transporters, cell wall reconstruction, etc. (Fig. 5a). GO-terms for down-regulated transcripts included water transport, malate metabolism, and regulation of ROS metabolic processes (Fig. 5b). Many underlying genes involved in membrane transporters, calcium signaling and protein kinases that were induced by cold stress are shown in Tables S5 and S6. These results suggest that the cell membrane, cell homeostasis, and calcium signaling play important roles in the early response to cold stress. GO terms related to flavonoid biosynthetic, ketone biosynthetic, and phenylpropanoid metabolic processes indicate that these secondary metabolites might be an integral part of sandalwood’s adaptation to cold stress. Interestingly, 36 over-represented GO terms were significantly enriched at 48 h/0 h (Fig. 5a). Among these, GO terms related to the response to endogenous stimuli, osmotic stress, hormones, and regulation of the defense response, were most dominant. The most notable are the enrichment of GO-terms related to the response to indole butyric acid or brassinosteroid (BR) stimulus, auxin polar transport, GA catabolism and the GA- or BR-mediated signaling pathway, as well as jasmonic acid (JA) biosynthesis. A number of DEGs related to hormones were found in the list of DEGs (Table S7). These suggest that trends in the expression of genes regulating cold response-related processes are enhanced as cold treatment proceeds, allowing sandalwood plants to adapt to environmental variations in which phytohormones play an important role. Based on this assessment, the transcriptomic data was divided into two groups, representing early (12, 24 h) and late (48 h) responses in *S. album*.

According to the KEGG function annotations, a total of 12, 22, and 10 pathways that were significantly enriched (Qvalue ≤ 0.05) were identified at 12 h/0 h, 24 h/0 h, and 48 h/0 h, respectively (Table 2). The “pyruvate metabolism”, “starch and sucrose metabolism”, and “carbon fixation in photosynthetic organisms” pathways that are involved in plastid photosynthesis were predominant in all three pairwise comparisons, suggesting that these pathways play important roles in the cold stress response. DEGs associated with membrane damage and cell construction were significantly enriched, including those involved in linoleic acid and fatty acid metabolism, the biosynthesis of unsaturated fatty acids, and other processes. As shown in Table 2 and Fig. S4, a number of genes in the “plant hormone signal transduction” pathway changed significantly. In addition, we noticed that monoterpenoid, diterpenoid or terpenoid backbone biosynthesis were significantly enriched in early and late responses.

### Photosynthesis under cold treatment

To further explore the effect of cold stress on photosynthesis, a total of 98 DEGs associated with the KEGG photosynthesis pathway in the KEGG database were detected (Table S8). The expression of these genes was classified into eight groups (Fig. S5). Genes in clusters 2, 3, and 8 were persistently up-regulated, the majority of which were induced by cold, whereas the expression of genes in cluster 4 was down-regulated. Interestingly, genes in clusters 1 and 6 were down-regulated at 12 h, but were once again induced at 24 h. These results indicate that diverse activation and repression of gene expression is associated with the photosynthesis of sandalwood plants exposed to cold treatment.

Among these DEGs, 23 out of the 32 induced transcripts were annotated as malate dehydrogenases (NADP-MDHs), implying that carbon dioxide fixation might be enhanced under cold treatment. Genes coding for electron transporters of photosystem I (PSI) and photosystem II (PSII), PSI P700 chlorophyll *a* apoprotein A2 (PsaB) and PSII CP47 chlorophyll apoprotein (PabB), and oxygen-evolving enhancer protein 2 (OEE2), a key protein of the photosynthetic light reaction in PSII, were induced by cold. Several genes encoding phytochrome-interacting factor 3 (PIF3), which is involved in the light-responsive transcriptional network, accumulated persistently. In contrast, two light-harvesting complex II chlorophyll *a*/*b* binding (LHCB) protein-coding genes declined sharply at 12 h, but accumulated significantly at 24 h and 48 h. Taken together, these results suggest that photoprotection, to some extent, increased in response to cold damage. In contrast, the expression of genes annotated as phosphoenolpyruvate carboxylase (PEPC), fructose-bisphosphate aldolase (FBPA), triosephosphate isomerase (TPI), and ribulose-phosphate 3-epimerase (RPE), was persistently down-regulated, suggesting that carbohydrate metabolism had weakened during cold stress.

### Response of genes involved in terpene biosynthesis under cold treatment

**Figure 6:**
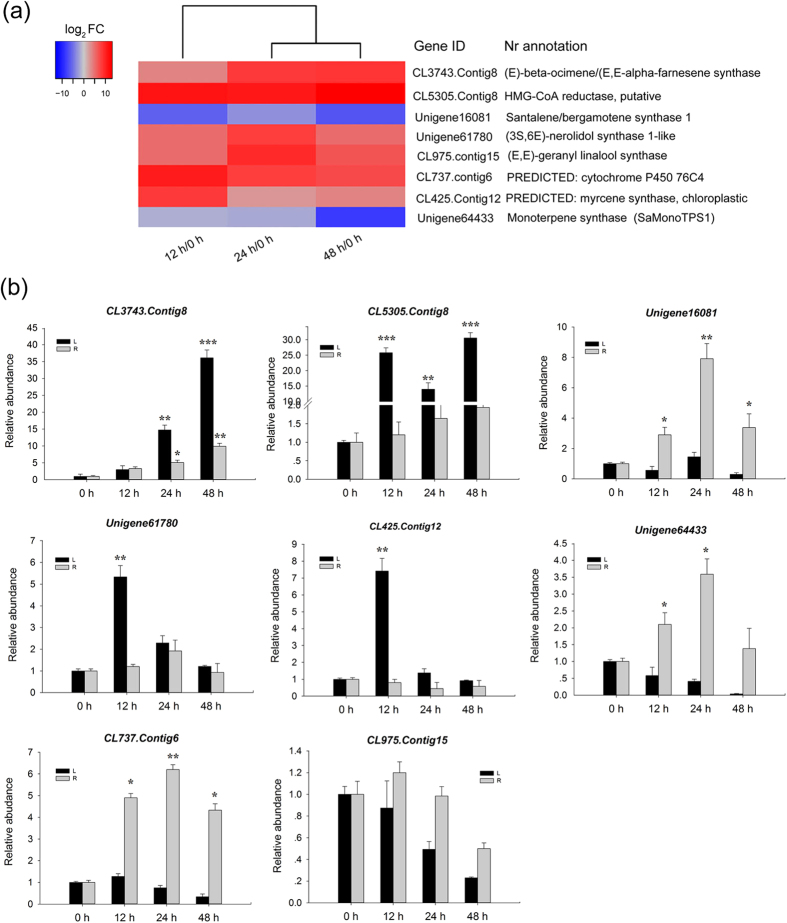
Differentially expressed genes involved in terpenoid biosynthesis in response to cold stress. (**a**) Heat map of changes of transcript abundance for eight genes. (**b**) qRT-PCR determination of transcript levels of these genes in leaves and roots following cold treatment (4 °C). Three measurements were averaged from the results of three replicated experiments and statistically treated using a *t*-test. *P ≤ 0.05 > 0.005; **P ≤ 0.005 < 0.001; ***P < 0.001. L, leaves. R, roots.

All terpenoids are synthesized through two independent pathways: the cytosolic mevalonic acid (MVA) pathway and the plastidial methyerythriol phosphate (MEP) pathway^27^. In this study, 51 unigenes were found to be potentially related to terpenoid backbone biosynthesis. Among these, at least one unigene from the transcriptome database of *S. album* leaves corresponded to the enzyme participating in core biosynthetic steps of the MVA and MEP pathways (Table S9). These unigenes were expressed to varying degrees, but there were no significant differences among them at 12, 24 or 48 h of cold stress compared to the control except for several homologs of the 3-hydroxy-3-methylglutaryl coenzyme A (HMG-CoA) reductase gene, a first rate-limiting enzyme in the MVA pathway. This gene had log2Ratio >10 values in treated leaves than in the control (CL5305.Contig8). More importantly, of the 41 transcripts encoding terpenoid synthases, seven genes were differentially expressed, including five up-regulated and two down-regulated by cold stress (Fig. 6a).

The full lengths of these seven genes besides one *HMG*-*CoA* were obtained by RACE. The sequences of two genes (unigene64433 and CL737.Contig6) were identical to *SaMonoTPS1* and *S. album bergamotene oxidase (SaCYP76F38*) submitted to NCBI (accession No. EU798692 and KC533715, respectively). The remaining six genes are being functionally characterized. We further measured the accumulation of these DEGs in leaves and roots under cold treatment using qRT-PCR. The expression patterns obtained by qPCR in leaves were in agreement with those inferred by the RNA-seq data (Fig. 6b). One gene encoding (*E*)-β-ocimene/(*E*,*E*)-α-farnesene synthase (BOS/AFS) was persistently induced in leaves, but increased slowly in roots in response to cold stress. Strikingly, three genes annotated as santalene/bergamotene synthase 1 (SS/BS), SaMonoTPS1, and SaCYP76F38 were repressed in leaves, but significantly up-regulated in roots in response to cold stress, suggesting that organ-specific terpenes might be involved in the response of *S. album* to cold stress. The level of expression of putative (*3* *S*,*6E*)-*nerolidol synthase 1*-*like* and *myrcene synthase* peaked significantly at 12 h of cold stress in leaves. These results confirmed that terpenoid synthase genes play a role in the cold stress response of *S. album*.

### Identification, isolation and characterization of cold-induced transcription factors

**Figure 7:**
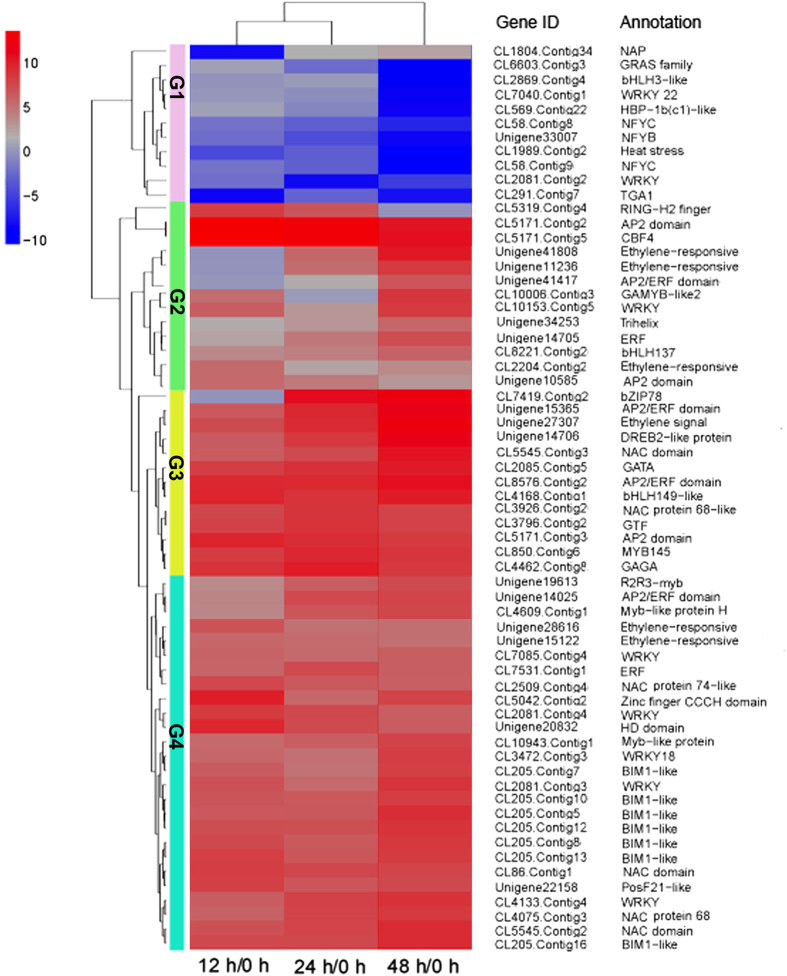
Hierarchically clustered heat map of cold regulatory transcript factor (TF) genes. The differentially expressed TFs were clustered into four groups (G1, G2, G3, and G4).

A total of 15 TF families consisting of 63 DEGs were identified (Table S10). Following cluster analysis, these TFs were clustered into four groups, i.e., G1, G2, G3, and G4 (Fig. 7). Strikingly, most of these TFs were up-regulated in the three pairwise comparisons, as shown in G2, G3, and G4, revealing that transcriptional activation, but not repression, was significantly involved in the cold stress response. DEGs in the AP2/ERF family were the most abundant. Of these, transcripts of three genes coding for an AP2 domain class TF, one for CBF4, and two for ethylene-responsive TFs, increased dramatically at 12 h and decreased gradually until 48 h of cold stress, suggesting that these TFs might play crucial roles in the early cold response. One zinc finger CCCH domain protein-coding transcript accumulated significantly, with a similar expression pattern as the AP2 domain class of TFs described above. Members of *MYB, bHLH* (basic helix-loop-helix), *WRKY, NAC* (NAM, ATAF1/2, CUC2), and *bZIP* families of TFs exhibited cold-inducible expression. Furthermore, a decline in the transcripts of developmental regulators was observed, including GRAS and NFY.

**Figure 8:**
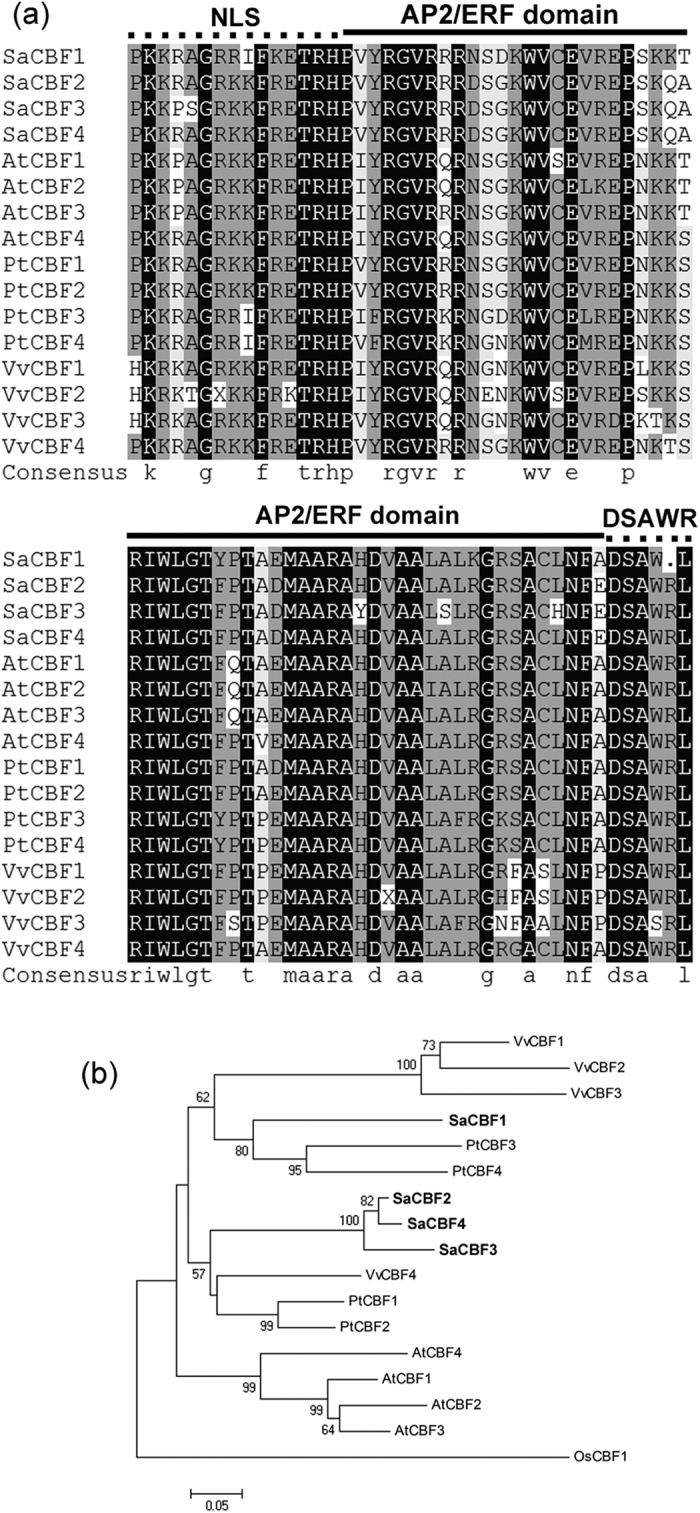
Comparison of representative C-repeat binding factor (CBF) protein family members in sandalwood, Arabidopsis (*Arabidopsis thaliana* (L.) Heynh.), poplar (*Populus trichocarpa* Torr. & A. Gray), and grape (*Vitis vinifera* L.). (**a**) Alignment of AP2 and flanking region of CBF factors. The AP2/ERF domain, putative nuclear localization signal (NLS), and DSAWR motif are marked with a solid line and a dotted line, respectively. (**b**) A phylogenetic analysis of sandalwood, Arabidopsis, poplar, and grape proteins, based on minimum evolution, was performed with the full-length protein sequences using OsCBF1 as an out-group. Sequences were aligned using four sandalwood CBF proteins: SaCBF1 (KX009414), SaCBF2 (KX009415), SaCBF3 (KX009416), and SaCBF4 (KX009417), four poplar CBF proteins, PtCBF1 (JGI666968), PtCBF2 (JGI346104), PtCBF3 (JGI548519), and PtCBF4 (JGI63608), four Arabidopsis CBF proteins, AtCBF1 (ABV27072.1), AtCBF2 (ABV27106.1), AtCBF3 (ABV27138.1), and AtCBF4 (ABV27155.1), and four grape CBF proteins, VvCBF1 (AIL00526.1), VvCBF2 (AIL00632.1), VvCBF3 (AIL00730.1), and VvCBF4 (AIL00786.1). CBF proteins were initially aligned using CLUSTALX (v1.83) and used to conduct phylogenetic analyses using *MEGA* version 4. A phylogenetic tree was constructed using the neighbor-joining method on 1,000 bootstrap replications. Bootstrap percentages are shown on the dendrogram branch points.

The full lengths of nine genes were obtained by RACE. Homology searches showed that these nine proteins are members of the AP/ERF domain and ANK superfamilies, and the bHLH family. Based on sequence similarities via BLAST in NCBI, these genes were designated as *SaCBF1, SaCBF2, SaCBF3, SaCBF4, SaERF017, SaERF109*-*like, SaRAP2*.*4, SaC3H29*, and *SaICE1* (Table S11). The full-length sequences of *SaCBF1* encode small proteins 215 amino acids long while SaCBF2 to 4 contain 220 amino acids. Alignment of the deduced amino acid sequences of *Sa*CBF1 to 4 with those of grape CBF1 to 4, poplar CBF1 to 4, and Arabidopsis CBF1 to 4 demonstrated significant similarities in the AP2/ERF binding domain and the CBF signature sequences between species (Fig. 8a). This result suggests that sandalwood CBFs share remarkable similarities at the amino acid sequence level with known CBF/DREB proteins in other plants and carry critical amino acids that are needed for binding to the CRT elements in the target genes. A phylogenetic analysis of the deduced protein sequences of the four sandalwood *CBF* genes with Arabidopsis CBF1 to 4 proteins, poplar CBF1 to 4 proteins, and grape CBF1 to 4 proteins showed that sandalwood CBF2 and 4 were closely grouped, whereas SaCBF1 was more closely related to poplar CBF1 and 3 (Fig. 8b).

**Figure 9:**
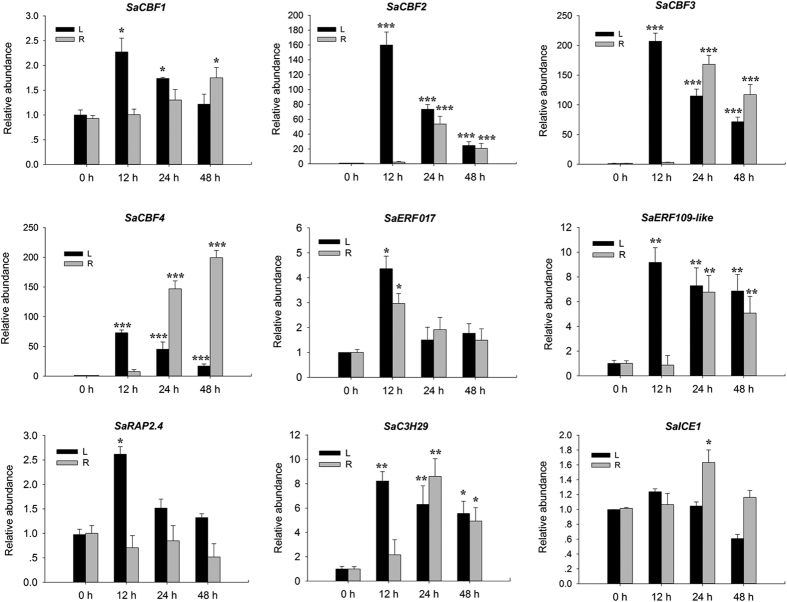
Expression patterns of cold-inducible genes shown by qRT-PCR following cold stress (4 °C) treatment. Three measurements were averaged from the results of three replicated experiments and statistically treated using a *t*-test. *P ≤ 0.05 > 0.005; **P ≤ 0.005 < 0.001; ***P < 0.001. L, leaves. R, roots.

The expression patterns of genes in leaves obtained by qPCR were in agreement with those inferred by the RNA-seq data, which further confirmed the reliability of the RNA-seq dataset. S*aCBF1* in leaves and roots was slightly up-regulated in response to cold stress (Fig. 9). However, transcripts of *SaCBF2* to *4* accumulated dramatically at 12 h in leaves, with a more than 50-fold increase compared to the control. Similarly, the expression levels of *SaCBF 2* to *4* peaked in roots at 24 h of cold treatment, suggesting that leaves were more susceptible to cold stress than roots. It is likely that leaves were directly exposed to cold air and responded quickly to cold stress more than roots, which were immersed in, and thus somewhat protected by, soil. The expression of *SaERF017, SaERF109*-*like, SaRAP2*.*4, SaC3H29*, and *SaICE1* exhibited similar expression patterns as *SaCBF*s over the 48-h time series of cold stress, but the relative expression ratios of these TFs were less than 10.

### Novel genes induced by cold stress in *S. album* leaves

As shown in Table S3, approximately 51% genes of all DEGs were found to not have any annotation (*ca*. 2000 genes) or to only have an Nr annotation (250 genes). These were considered to be novel genes in response to cold stress. Of these, about 80% genes were activated by cold. A Venn diagram showed that 894, 778, and 883 novel genes were induced at 12, 24, and 48 h of cold treatment, respectively (Fig. S6a). Moreover, 184 genes were commonly expressed at all three time points compared with 0 h. Notably, there were 442, 284, and 485 genes that were uniquely induced at 12 h/0 h, 24 h/0 h, and 48 h/0 h, implying that these genes might play important roles at different time points of cold stress. These novel genes could be divided into 27 clusters, and clusters 1 to 8 were significantly different, showing *P* values less than 0.05 (Fig. S6b). The expression profile of genes in these significant clusters showed that some genes accumulated persistently throughout the 48-h period of cold stress (Fig. S6ci,ii). In contrast, genes were activated at 12 h, but were then completely repressed as the cold stress period was lengthened to 48 h (Fig. S6ciii–v). Interestingly, genes were induced at 12 h, repressed at 24 h, but further activated at 48 h of cold stress (Fig. S6cvi,vii). Moreover, the expression level of many induced genes remained relatively stable from 12 to 48 h of cold stress (Fig. S6cviii). Thus, the potential response mechanism of cold stress is complex in *S. album*.

## Discussion

*S. album* is an economically important plant species because of its highly valued essential oils used in the perfumery industry. Most research has focused on the identification of sandalwood oil components and on the functional characterization of genes coding for enzymes that generate major constituents^22^,^23^,^24^. In recent years, RNA-seq has emerged as a powerful and cost-efficient tool for global analyses of transcriptomic changes when genomic information is not available. The molecular characterization of the biosynthesis of oil production in *S. album* has been conducted based on transcriptome data from heartwood tissues being particularly rich in sequiterpenoids^26^,^27^,^28^. In this study, we provided a transcriptome database of approximately 10.0 GB of data from *S. album* leaves and assembled into 135,938 unigenes, more than half of which were annotated against the NCBI NR database. This work will provide an important public genomic resource and facilitate further functional genomic studies of this essential oil-bearing plant.

Although plants are exposed to fluctuating external temperatures, they can enhance their cold tolerance by modulating gene induction and transcription in response to cold stress^4^. A total of 3075 cold-inducible and 1349 cold-repressed genes were identified in the present study. This predominance of gene induction during cold stress indicates a continuous adaptation over an extended period of time. More than 50% of the *S. album* DEGs had no annotated homologs in the Nr database and these may represent cold-responsive genes with homologs that have not been identified in previous studies with other plants or that are specific to *S. album*. Further research of these genes may reveal new mechanisms of cold-stress responses for *S. album* plants. In addition, many metabolites that contribute to the induction of stress tolerance have long been linked to stress responses^36^. An increase in the contents of MDA, proline and soluble sugars, as well as EL have been demonstrated in cold-tolerant plants such as *Vitis amurensis* Rupr., wheat, and wild tomato^9^,^37^. Consistent with those reports, an increase in MDA, EL, proline and solute sugar content were observed over the 48-h time course of cold stress, indicating that osmolytes might protect plant cell membranes and increase membrane stabilization during cold-induced dehydration in *S. album*. Taken together, these results suggest that *S. album* have the ability to withstand the stress of short-period when exposed to cold.

All aspects of photosynthesis including the light reactions, the Calvin cycle, and photorespiration can be affected by low temperature stress^38^,^39^. The cessation of growth resulting from cold stress reduces the capacity for energy utilization and causes feedback inhibition of photosynthesis^40^. In the present study, significant GO terms for “malate metabolic process” and “carbon utilization” appeared in the early response but were not detected in the late response to cold stress. Although photo-protection was activated during cold stress, cold-induced photo-inhibition finally occurred as the period of cold treatment was prolonged, as confirmed by our physiological analysis. This supports the fact that photosynthetic efficiency is negatively affected by cold stress. ROS accumulation is connected to decreased photosynthetic efficiency, and is caused by a failure in the electron transfer reaction of PSI^41^. The enzymatic antioxidant system is one of the protective mechanisms employed to eliminate or reduce ROS and increase a plant’s capacity for stress tolerance under abiotic stress^42^,^43^. Increased SOD and POD activity was detected in our study. The significant enrichment of GO terms related to increased expression of cold-responsive genes, including a response to oxygen-containing compounds or alcohol, and regulation of a plant-type hypersensitive response, were found in the early and late cold responses. This indicates that a dynamic balance in ROS formation and removal from cells to protect them against oxidative damage occurred during cold stress. Moreover, evidence in Arabidopsis showed that flavonoids as antioxidants in photo-protection played important roles in freezing tolerance^44^. Our identification of robust GO terms related to flavonoid biosynthesis implied that flavonoids might be involved in reducing the oxidative damage caused by cold stress. Importantly, the GO terms of up-regulated transcripts related to “response to wounding”, “regulation of defense”, and “regulation of immune response” were detected in the late cold response (Fig. 5a). Therefore, it can be hypothesized that *S. album* plants recruit a defense mechanism to increase cold tolerance when exposed to a prolonged period of cold.

Terpenes are a very large and structurally diverse group of secondary metabolites which are widely distributed in the plant kingdom^45^. Terpene biosynthesis in plants is of great interest because terpenoids play important roles in enhancing biotic or abiotic stress tolerance^46^. Research suggested that plants might enhance their tolerance to environmental stress (drought, high light intensity or high temperature) by increasing the emission of isoprenes or monoterpenes^47^. Evidence showed that sesquiterpenes, (*E*)-β-farnesene and (*E*)-α-bergamotene quench ozone but could not protect wild *Nicotiana attenuata* Torr. ex S. Watson from ozone, UVB light, and drought stresses^48^. Previous studies and our study indicate that transcripts generating terpenoids were expressed in multiple tissues of sandalwood, including leaves, heartwood, and roots. However, the molecular regulation of santalol biosynthesis in sandalwood is largely unknown. A foliar application of methyl jasmonate caused a higher expression level of *HMGR* and the farnesyl diphosphate synthase gene in leaves and stems of treated *S. album* seedlings compared to untreated plants at 4 h, but expression of *cytochrome P450* showed no change in both leaves and stems^29^. In the present study, several homologs of *HMGR* were dramatically up-regulated in leaves by cold stress, implying that this key rate-limiting enzyme in MVA is likely to play an important role in the response of *S. album* to external stress. To date, all terpenes that have shown enhanced abiotic stress tolerance were synthesized in plastids^45^. Under stress, photosynthetic efficiency is closely associated with the accumulation of ROS which constrains the electron transfer reaction of PSI^41^. As evidenced in our study, cold stress led to a decrease in photosynthetic efficiency in *S. album*. We speculate that these putative terpene synthase genes may play a role in protection against localized oxidative stress in plastids. A sesquiterpene-rich essential oil is very abundant in the heartwood and roots of sandalwood^21^,^23^. High-level accumulation of *SS*/*BS, SaMonoTPS1*, and *SaCYP76F38* in sandalwood roots implies that these genes play a role in protecting sandalwood from cold stress damage. In contrast, high expression of *BOS*/*AFS* in leaves was detected, suggesting that there is likely a different protection mechanism in different organs of *S. album* under cold stress. Functional roles for terpene synthase genes need to be further investigated in *S. album*.

Research in recent years has indicated that hormones play important roles in regulating plant freezing tolerance by either CBF-dependent or -independent pathways^19^. Our robust GO term annotation and KEGG pathway analysis showed the involvement of auxin, cytokinin, GA, ET, JA, BRs, ABA and salicylic acid in the response of *S. album* to cold stress (Fig. 5; Fig. S4), indicating that a complex regulatory network of hormones exists. What most deserves to be mentioned is the role of GA in response to cold stress. Exposure of Arabidopsis seedlings to cold stress stimulated the accumulation of DELLA proteins, suggesting that a link between CBFs and GA in cold stress-induced growth retardation exists^49^,^50^. Similarly, the overexpression of *GhDREB1* and *CbCBF* in *Gossypium hirsutum* L. and *N. tabacum* L., respectively, caused a reduction of bioactive GA^51^,^52^. Other studies provide evidence that GA signaling components can affect the low temperature stress responses of plants^53^. In our study, up-regulated *GA2 oxidases (GA2ox*) and the GO term “GA mediated signaling pathway” were detected, highlighting the fact that GA metabolism and signaling are involved in the cold-stress response of *S. album* plants. Conversely, one *GA20 oxidase (GA20ox*, CL2514.Contig1) showed increased expression, with a log_2_ ratio of more than 8 compared to the control at 24 and 48 h (Table S7). It is worth exploring the significance of genes in the biosynthesis of GA in response to cold stress in the future.

Transcriptome profiling indicates that multiple regulatory pathways other than the CBF cold response pathway are activated during cold acclimation^54^. Park *et al*.^12^ found that there are at least 17 cold-induced TFs with similar expression patterns as those of *CBFs* in Arabidopsis, including *ZF, CZF1, RAV1, MYB73, ZAT12, HSFC1, MYB44, ERF5*, etc.^12^. Transcriptomic analysis showed that several *RAP* and *ERF* homologs were up-regulated under cold (4 °C) and freezing (−4 °C) treatment in desert poplar (*Populus euphratica* Oliv.)^55^. Our study indicates that *SaCBF1* to *4*, besides *SaERF017, SaERF109*-*like, SaRAP2*.*4*, and *SaC3H29*, play important roles in the regulation of the cold stress response in sandalwood. This is in agreement with the known fact that a complex regulatory network exists besides the CBF pathway in response to cold stress^4^. However, Park *et al*.^12^ thought that the increase in freezing tolerance that occurs with cold acclimation is only partially dependent on the CBF-CRT/DRE regulatory module in Arabidopsis. Since cold stress leads to cellular dehydration and an elevation of salt concentration, a dynamic crosstalk exists between the cold stress-signaling pathway and other signaling systems associated with dehydration and salt stress^56^,^57^. This study demonstrated that *SaCBF2* to *4* was strongly responsive to cold stress in the leaves and roots of sandalwood, although the expression patterns of other TFs, *SaERF017, SaERF109*-*like, SaRAP2*.*4*, and *SaC3H29* were similar to those of *SaCBF*s (Fig. 8, Table S9). This suggests that *SaCBF*s are critical contributors to the cold regulatory mechanism in *S. album* and that other cold-inducible TFs might be involved in the regulation of *COR* gene expression.

The present study generated a robust and well-annotated transcriptome for the cold stress response in sandalwood leaves. Based on the comprehensive physiological and transcriptomic analyses, when combined with RACE and qRT-PCR, our study provides fundamental knowledge related to the physiological changes and gene regulatory networks that operate when a sandalwood seedling is exposed to cold treatment. After a seedling perceives low temperature through cell membrane receptors, cold signal transduction induces changes to cellular metabolism, including calcium influx, the activation of Ca^2+^-dependent protein kinase cascades, the accumulation of cryoprotectants, proline, soluble sugar, and the antioxidants SOD and POD. Photosynthetic efficiency is negatively affected by cold stress. Terpenoid synthase genes were also involved in the cold stress response. Phytohormones play important roles in the response to cold stress, although further validation is required. The CBF-dependent signaling pathway might play a crucial role in the regulation of cold tolerance in sandalwood. Thus, a hypothetical model for sandalwood cold response networks has been proposed (Fig. S7). The functions of candidate genes involved in the regulation of cold signaling and cold-resistance genes will be verified in the future. This study contributes to uncovering the molecular background of *S. album*’s defense response to cold stress that will allow for the planting area to be expanded and for improving the cold tolerance of sandalwood by genetic engineering. Moreover, the finding that terpene synthase genes are induced by cold stress provides a clue as to how to stimulate santalol biosynthesis and accumulate essential oil in this species.

## Methods

### Plant material

Fully ripe *S. album* seeds collected from 20-year-old trees growing in SCBG were germinated according to our previously described method^58^. Seedlings were transplanted to 2-L pots filled with a mixture of sand, peat, and soil (3:1:1, v/v/v) and fertilized with 10 mL of 5% fertilizer whose optimum N: P: K ratio was 2.1: 1: 0.4 for each pot per week. Plants were cultivated in a greenhouse under a natural photoperiod (Feb-May, 2015, Guangzhou, China) with a day/night temperature of about 20–28 °C, 70–80% relative humidity, and a photosynthetic photon flux density (PPFD) of 140–260 μmol m^−2^ s^−1^. Seedlings were obtained after three months of culture. The host plant, *Kuhnia rosmarnifolia* Vent., which is a potential pot host for *S. album* in China^34^, was assembled to encourage host-parasite infection following our previous method^59^. Plants were watered twice daily with tap water. Pots were randomly moved every week to minimize position effects.

### Cold treatment and sample collection

Five and a half month old seedlings with uniform growth were transferred to a phytotron under standardized growth conditions (16-h photoperiod, 28/23 °C (day/night), PPFD = 80 μmol m^−2^ s^−1^) and acclimated for two weeks prior to cold treatment. Seedlings were subjected to 4 °C for 0, 12, 24, and 48 h, serving as the cold stress treatments, under the same light conditions in the phytotron. The first and second fully expanded leaves were harvested immediately after the completion of each period of cold exposure, quickly frozen in liquid nitrogen, and stored at −80 °C until use. Physiological and biochemical indexes were measured using leaves at the same position as those used for RNA sample collection. The samples from five individuals were pooled for each time point and the collection of leaf material was repeated three times from different seedlings, serving as three independent biological replicates.

### Physiological analyses of cold-treated leaves from *S. album*

To analyze the physiological changes in *S. album* leaves under cold stress prior to the RNA-seq study, three indicators of the cold response were estimated: MDA and proline content, and EL. MDA content was determined by the thiobarbituric acid reaction^60^. Proline concentration was measured by the sulfosalicylic acid-acid ninhydrin method^61^. EL was estimated as previously described^62^. Electrical conductivity of the bathing solution (Lt) was determined by a digital conductometer DDS-11A (Leici Instrument Factory, Shanghai, China) and a last conductivity reading (L0) was obtained upon equilibration at 25 °C after the bathing solution with leaf tissues was autoclaved at 120 °C for 20 min. In addition, the level of total soluble carbohydrates was determined using the anthrone method with glucose as the standard^63^. All spectrophotometric measurements were performed using a Beckmann DU640 (MI, USA) spectrophotometer.

### Assay for antioxidative enzyme activities

To measure enzyme activities, 2 g of leaves were homogenized in 10 mL of 50 mM potassium phosphate buffer (pH 7.8) containing 4% polyvinylpyrrolidone (MW: 40,000). The homogenate was centrifuged at 12,000 rpm for 20 min and the supernatant was used for enzyme analyses at room temperature using a Beckmann DU640 spectrophotometer. All steps in the preparation of the enzyme extract were conducted at 0–4 °C. SOD activity was expressed on the basis of the inhibition of the photochemical reduction of nitro blue tetrazolium (NBT) at 560 nm^64^. The quantity of enzyme that reduced NBT by 50% in the dark was defined as one unit of SOD activity. POD activity was assayed following a method originally applied to *Glycyrrhiza uralensis*^65^. Absorbance was measured at 20-sec intervals at 460 nm for a total of 3 min. One unit of POD activity was defined as the increase by one unit per minute of the absorbance at 460 nm.

### Measurement of photosynthetic parameters

Indexes of photosynthesis including A, Gs, E, Ci, and R of the third and fourth fully expanded leaves of six-month-old seedlings exposed to cold for 0, 12, 24, and 48 h were measured under standardized conditions with a portable photosynthesis system (LI-6400, Li-COR Inc., Lincoln, NE, USA). The measurement conditions were: 150 μmol m^−2^ s^−1^ PPFD, 70% relative air humidity, and 400 μmol mol^−1^ ambient CO_2_ concentration. WUE was calculated as the ratio of A to E^66^. Measurements were made on five plants for each of the three biological replicates of each treatment.

### RNA isolation, library construction, and sequencing

Total RNA was extracted using Trizol (Invitrogen, Carlsbad, CA, USA) and treated with DNase I to remove genomic DNA contamination using the Turbo DNA-free Kit (Ambion, Austin, TX, USA). RNA was quantified using a NanoDrop ND-1000 spectrophotometer (Nanodrop Technologies, Wilmington, NC, USA). The quality of total RNA was determined using an Agilent 2100 Bioanalyzer (Agilent Technologies, Palo Alto, CA, USA). One cDNA library was constructed from total RNA of non-treated and treated leaf samples for *de novo* assembly of the sandalwood transcriptome using an Illumina TruSeq RNA Sample Preparation Kit (Illumina, San Diego, CA, USA). Also, a total of 12 libraries were separately generated from the control sample and from 12, 24, and 48 h cold-treated samples using the same protocol. Reads were generated in a 100 bp paired-end for *de novo* assembly using an Illumina Hiseq 2000 platform at the Beijing Genomics Institute (BGI). The libraries were sequenced with 50 bp single end reads for RNA-seq analysis (mRNA quantitative analysis) on the Illumina Hiseq 2000 platform.

### Data processing and analysis

Transcriptome *de novo* assembly was performed using Trinity^67^. Firstly, shorts reads were combined with certain a length of overlap to form longer fragments without N, known as contigs. These reads were then mapped back to contigs. Using paired-end reads, we detected contigs from the same transcript as well as the distances between these contigs. Next, we used Trinity to connect the contigs and obtained sequences that cannot be extended on either end, which were defined as unigenes. Finally, we assembled all the unigenes to form a single set of non-redundant unigenes using TGICL^68^. After clustering, the unigenes were divided into two classes: clusters and singletons. After assembly, BLASTx alignment was performed between unigenes and the protein databases with a cut-off E-value of 10^−5^, following this order of priority: Nr, Swiss-Prot, KEGG, COG and GO. The best alignment results were used to decide the sequence direction of unigenes.

Raw reads of FASTQ format from RNA-seq data of control and treated samples were first processed through in-house Perl scripts. In this step, clean reads were obtained by removing reads containing an adapter, reads in which unknown bases accounted for more than 10%, and low quality reads (i.e., where more than 50% of bases in a read had a quality value Q ≤ 5) from the raw data. Clean reads were aligned to the reference unigenes using Bowtie^69^. Gene expression levels were quantified by RSEM (RNASeq by Expectation Maximization)^70^, which computes maximum likelihood abundance estimates using the Expectation-Maximization (EM) algorithm for its statistical model, including the modeling of paired-end, variable-length reads, fragment length distributions, and quality scores, to determine which transcripts are isoforms of the same gene. Gene expression level was calculated with the FPKM method^71^. The number of transcripts after filtering among all samples was analyzed by a bar graph. A correlation between samples was performed based on FPKM results by calculating Pearson’s correlation for pairs of samples. Principal component analysis was performed to visualize the distance of the relationship between each sample using R statistical software (version 3.2.3)^72^.

DEGs under cold stress were screened using the Noiseq package with the following criteria: fold change ≥2 and deviation probability ≥0.8 at 12 h/0 h, 24 h/0 h, and 48 h/0 h, respectively^73^. Hierarchical clustering was performed for DEGs with a ≥8-fold change and deviation probability ≥0.8 in at least one of the three pairwise comparisons using the Manhattan distance matrix of the heatmap.2 function in the gplots package of the R environment. We then separately mapped induced or repressed DEGs to each term of the GO database (http://www.geneontology.org/) and found significantly enriched GO terms in cold-inducible and cold-repressed genes using a hypergeometric test^55^. The calculated *P* value underwent a Bonferroni correction until the threshold of the corrected *P* value was less than 0.05. GO terms fulfilling this condition were defined as significantly enriched GO terms to detect the main biological functions that DEGs exercise during cold stress.

### Screening and cloning of cold-induced transcription factors

In order to identify exactly which TF genes were highly expressed during cold stress, the predicted coding sequences of seven genes encoding AP2 domain-containing TFs, CBF4, RAP2-4-like, ethylene-responsive TF, and zinc finger CCCH domain-containing protein 29-like, in addition to one *inducer of CBF expression 1* and one *CBF*/*DREB*-*like* gene, were obtained from assembled unigenes. The SMARTer™ RACE cDNA Amplification Kit (Clontech Laboratories Inc., Takara Bio, Mountain View, CA, USA) was used to generate both 5′ and 3′ cDNA ends. The 5′ and 3′ RACE PCR products were purified by a Gel Extraction Kit (Dongsheng Biotech, Guangzhou, China), cloned into the pMD18-T vector (Takara Bio Inc., Dalian, China) and sequenced at the BGI. Full-length cDNAs were amplified with primers designed from 5′ and 3′ untranslated regions (UTR) by PCR using the LA PCR Amplification Kit (Takara Bio Inc.). All primers used to amplify 3′ and 5′ UTR regions and full-length cDNAs are listed in Table S12. The multiple alignments were used by DNAMAN8.0 (Lynnon Biosoft, San Ramon, CA, USA) and a phylogenetic tree was constructed by the neighbor-joining (NJ) method using MEGA 4.0^74^,^75^.

### qRT-PCR analyses

Total RNA extracted from *S. album* leaves and roots after 0, 12, 24, 48 h of cold stress were treated with DNase I to remove genomic DNA contamination using the TurboDNA-free Kit (Ambion, Austin, TX, USA). First-strand cDNA synthesis and purification followed our published method^58^. Gene-specific primers are listed in Table S13. Melting curve analysis was performed for each primer pair prior to further analyses. qRT-PCR reactions were performed with the SYBR Green Real Master Mix (Bio-RAD, Foster, CA, USA) in the ABI 7500 fast Real-Time PCR system (Applied Biosystems, Foster, CA, USA). The expression level was calculated using our protocol^58^. qRT-PCR results were obtained from three biological replicates and three technical repeats for each gene and sample.

## Additional Information

**How to cite this article**: Zhang, X. *et al*. Physiological and transcriptomic analyses reveal a response mechanism to cold stress in *Santalum album* L. leaves. *Sci. Rep.* **7**, 42165; doi: 10.1038/srep42165 (2017).

**Publisher's note:** Springer Nature remains neutral with regard to jurisdictional claims in published maps and institutional affiliations.

## Supplementary Material

Supplementary Files

Supplementary Table S3

Supplementary Tables S5-8,10

## Figures and Tables

**Table 1:** Alignment statistics result with reference gene for all samples.

Sample	Total reads (clean data)	Total base pairs	Total mapped reads	Perfect match	Mismatch	Unique match	Multi-position match	Total unmapped reads	Expressed unigenes
0 h_1	13,119,262	642,843,838	11,950,889	9,586,139	2,364,750	6,153,440	5,797,449	1,168,372	81,809
	(100.00%)	(100.00%)	91.09%	73.07%	(18.03%)	(46.90%)	(44.19%)	(8.91%)	
0 h_2	13,119,005	642,831,245	11,991,012	9,643,018	2,347,994	6,062,151	5,928,861	1,127,992	82,796
	(100.00%)	(100.00%)	(91.40%)	(73.50%)	(17.90%)	(46.21%)	(45.19%)	(8.60%)	
0 h_3	13,120,268	642,893,132	11,791,460	9,478,104	2,313,356	6,102,912	5,688,548	1,328,807	81,939
	(100.00%)	(100.00%)	(89.87%)	(72.24%)	(17.63%)	(46.52%)	(43.36%)	(10.13%)	
12 h_1	11,555,790	566,233,710	10,424,232	8,386,016	2,038,216	5,180,950	5,243,282	1,131,557	89,897
	(100.00%)	(100.00%)	(90.21%)	(72.57%)	(17.64%)	(44.83%)	(45.37%)	(9.79%)	
12 h_2	13,120,967	642,927,383	11,900,415	9,609,364	2,291,051	5,854,028	6,046,387	1,220,551	93,529
	(100.00%)	(100.00%)	(90.70%)	(73.24%)	(17.46%)	(44.62%)	(46.08%)	(9.30%)	
12 h_3	13,120,241	642,891,809	11,885,854	9,650,657	2,235,197	5,870,219	6,015,635	1,234,386	93,086
	(100.00%)	(100.00%)	(90.59%)	(73.56%)	(17.04%)	(44.74%)	(45.85%)	(9.41%)	
24 h_1	13,118,702	642,816,398	11,936,092	9,591,295	2,344,797	6,141,804	5,794,288	1,182,609	89,565
	(100.00%)	(100.00%)	(90.99%)	(73.11%)	(17.87%)	(46.82%)	(44.17%)	(9.01%)	
24 h_2	13,119,336	642,847,464	11,887,991	9,574,330	2,313,661	6,154,363	5,733,628	1,231,344	88,867
	(100.00%)	(100.00%)	(90.61%)	(72.98%)	(17.64%)	(46.91%)	(43.70%)	(9.39%)	
24 h_3	13,118,338	642,798,562	11,876,813	9,588,758	2,288,055	5,993,404	5,883,409	1,241,524	88,874
	(100.00%)	(100.00%)	(90.54%)	(73.09%)	(17.44%)	(45.69%)	(44.85%)	(9.46%)	
48 h_1	13,119,184	642,840,016	11,962,081	9,623,410	2,338,671	5,697,962	6,264,119	1,157,102	84,528
	(100.00%)	(100.00%)	(91.18%)	(73.35%)	(17.83%)	(43.43%)	(47.75%)	(8.82%)	
48 h_2	13,119,012	642,831,588	11,997,487	9,726,911	2,270,576	5,792,056	6,205,431	1,121,524	88,133
	(100.00%)	(100.00%)	(91.45%)	(74.14%)	(17.31%)	(44.15%)	(47.30%)	(8.55%)	
48 h_3	13,119,957	642,877,893	11,978,040	9,635,630	2,342,410	5,838,364	6,139,676	1,141,916	86,367
	(100.00%)	100.00%	(91.30%)	(73.44%)	(17.85%)	(44.50%)	(46.80%)	(8.70%)	

**Table 2:** Significantly enriched gene pathways involving differentially expressed genes following cold stress treatment.

#	Pathway ID	Pathway	DEGs with pathway annotation	Q value
**0 vs 12 h**				
1	ko00620	Pyruvate metabolism	35 (5.53%)	3.47E-13
2	ko00591	Linoleic acid metabolism	16 (2.53%)	8.95E-11
3	ko00710	Carbon fixation in photosynthetic organisms	26 (4.11%)	8.95E-11
4	ko00053	Ascorbate and aldarate metabolism	19 (3%)	1.14E-07
5	ko00910	Nitrogen metabolism	14 (2.21%)	6.11E-04
6	ko00592	α-Linolenic acid metabolism	13 (2.05%)	8.20E-04
7	ko00500	Starch and sucrose metabolism	30 (4.74%)	2.29E-03
8	ko01100	Metabolic pathways	167 (26.38%)	4.81E-03
9	ko00380	Tryptophan metabolism	8 (1.26%)	1.28E-02
10	ko00410	β-Alanine metabolism	9 (1.42%)	3.26E-02
11	ko00340	Histidine metabolism	10 (1.58%)	4.12E-02
12	ko00280	Valine, leucine and isoleucine degradation	12 (1.9%)	4.12E-02
**0 vs 24 h**				
1	ko00620	Pyruvate metabolism	36 (6.24%)	3.14E-15
2	ko00053	Ascorbate and aldarate metabolism	22 (3.81%)	1.32E-10
3	ko00710	Carbon fixation in photosynthetic organisms	23 (3.99%)	2.95E-09
4	ko00591	Linoleic acid metabolism	11 (1.91%)	3.45E-06
5	ko00592	α-Linolenic acid metabolism	15 (2.6%)	1.47E-05
6	ko00380	Tryptophan metabolism	11 (1.91%)	5.32E-05
7	ko00071	Fatty acid metabolism	15 (2.6%)	7.20E-05
8	ko00640	Propanoate metabolism	13 (2.25%)	1.10E-04
9	ko00410	β-Alanine metabolism	12 (2.08%)	2.36E-04
10	ko00310	Lysine degradation	12 (2.08%)	6.02E-04
11	ko00280	Valine, leucine and isoleucine degradation	15 (2.6%)	6.02E-04
12	ko00330	Arginine and proline metabolism	14 (2.43%)	2.64E-03
13	ko00902	Monoterpenoid biosynthesis	5 (0.87%)	2.95E-03
14	ko01100	Metabolic pathways	152 (26.34%)	4.54E-03
15	ko00910	Nitrogen metabolism	11 (1.91%)	4.54E-03
16	ko00340	Histidine metabolism	11 (1.91%)	4.65E-03
17	ko00500	Starch and sucrose metabolism	26 (4.51%)	4.81E-03
18	ko01110	Biosynthesis of secondary metabolites	76 (13.17%)	5.13E-03
19	ko00561	Glycerolipid metabolism	12 (2.08%)	1.65E-02
20	Ko00040	Pentose and glucuronate interconversions	679 (1.39%)	1.65E-02
21	ko04075	Plant hormone signal transduction	43 (7.45%)	2.67E-02
22	ko00904	Diterpenoid biosynthesis	5 (0.87%)	2.75E-02
**0 vs 48 h**				
1	ko00710	Carbon fixation in photosynthetic organisms	24 (2.56%)	2.00E-05
2	ko00620	Pyruvate metabolism	27 (2.88%)	2.22E-04
3	ko04626	Plant-pathogen interaction	85 (9.06%)	7.69E-04
4	ko00902	Monoterpenoid biosynthesis	7 (0.75%)	1.90E-03
5	ko00904	Diterpenoid biosynthesis	9 (0.96%)	2.66E-03
6	ko00592	α-Linolenic acid metabolism	15 (1.6%)	3.81E-03
7	ko01040	Biosynthesis of unsaturated fatty acids	12 (1.28%)	1.85E-02
8	ko00270	Cysteine and methionine metabolism	14 (1.49%)	4.74E-02
9	ko00900	Terpenoid backbone biosynthesis	13 (1.39%)	4.78E-02
10	ko01100	Metabolic pathways	228 (24.31%)	4.84E-02
