# Supplementary material for: Physiological and transcriptomic analyses reveal a response mechanism to cold stress in Santalum album L. leaves
Source: Sci Rep. 2017 Feb 7;7:42165. doi: 10.1038/srep42165 (PMC5294638; doi:10.1038/srep42165)
Supplement: Supplementary Files [file srep42165-s1.pdf]

Supplementary information

Physiological and transcriptomic analyses reveal a response mechanism to cold stress in *Santalum album* L. leaves

Xinhua Zhang[1*], Jaime A. Teixeira da Silva[2*], Meiyun Niu[1], Mingzhi Li[1], Chunmei He[1], Jinhui Zhao[1], Songjun Zeng[1], Jun Duan[1], Guohua Ma[1]

[1] Key Laboratory of Plant Resources Conservation and Sustainable Utilization, South China Botanical Garden, Chinese Academy of Sciences, Guangzhou, China

[2] P. O. Box 7, Miki cho post office, Ikenobe 3011-2, Kagawa-Ken, 761-0799, Japan

[*]To whom correspondence should be addressed: xhzhang@scib.ac.cn; jaimetex@yahoo.com

Table S1. Summary of output statistics from *S. album*

Samples	Total raw reads	Total clean reads	Total clean nucleotides (nt)	Q20 (%)	N (%)	GC (%)
Leaves	100,445,724	97,349,766	9,734,976,600	95.40	0.00	47.03

Table S2 Correlation coefficient between paired samples

Sample	0 h_1	0 h_2	0 h_3	12 h_1	12 h_2	12 h_3	24 h_1	24 h_2	24 h_3	48 h_1	48 h_2	48h_3
0 h_1	1											
0h_2	0.9327	1										
0h_3	0.9825	0.9229	1									
12h_1	0.9418	0.9297	0.9074	1								
12h_2	0.9118	0.9081	0.8016	0.9372	1							
12h_3	0.957	0.9302	0.9267	0.9896	0.9404	1						
24h_1	0.914	0.9217	0.8836	0.9348	0.9367	0.9384	1					
24h_2	0.9604	0.9477	0.9505	0.9363	0.889	0.9545	0.9615	1				
24h_3	0.9549	0.9307	0.9486	0.9219	0.8667	0.9423	0.9541	0.9824	1			
48h_1	0.8855	0.8715	0.877	0.8805	0.8482	0.8956	0.9196	0.9305	0.9564	1		
48h_2	0.7699	0.8763	0.7448	0.8341	0.8802	0.8304	0.9291	0.8709	0.8778	0.9219	1	
48h_3	0.8579	0.8876	0.8506	0.8557	0.8716	0.8748	0.9211	0.9372	0.9171	0.9314	0.9268	1

Values were highlighted in green among biological replicates at each time point.

Table S4 Significant gene ontology (GO) terms for cold-inducible and cold-repressed genes under biological processes

GO ID	GO term	Percentage	Corrected P-value
**GO terms for cold-inducible genes**			
**0 vs 12 h**			
GO:0031408	oxylipin biosynthetic process	2.90%	2.26E-07
GO:0005992	trehalose biosynthetic process	3.10%	5.29E-07
GO:0046351	disaccharide biosynthetic process	3.40%	6.86E-07
GO:0005991	trehalose metabolic process	3.10%	2.98E-06
GO:0006108	malate metabolic process	2.20%	4.29E-06
GO:0015804	neutral amino acid transport	3.10%	7.11E-06
GO:0009312	oligosaccharide biosynthetic process	3.40%	1.02E-05
GO:0015976	carbon utilization	1.80%	0.00017
GO:0006833	water transport	2.90%	0.00068
GO:0042044	fluid transport	2.90%	0.00068
GO:0009719	response to endogenous stimulus	16.40%	0.00082
GO:0015800	acidic amino acid transport	1.30%	0.00104
GO:0010243	response to organic nitrogen	5.80%	0.00154
GO:0005984	disaccharide metabolic process	5.20%	0.00244
GO:0055081	anion homeostasis	1.60%	0.00407
GO:0043067	regulation of programmed cell death	5.20%	0.00565
GO:0006865	amino acid transport	4.70%	0.00614
GO:0006633	fatty acid biosynthetic process	4.30%	0.00638
GO:0009311	oligosaccharide metabolic process	5.40%	0.01606
GO:0016053	organic acid biosynthetic process	11.20%	0.02005
GO:0046394	carboxylic acid biosynthetic process	11.20%	0.02005
GO:0009725	response to hormone stimulus	14.30%	0.02066
GO:0010941	regulation of cell death	5.20%	0.02382
GO:0070588	calcium ion transmembrane transport	1.60%	0.02959
GO:0043069	negative regulation of programmed cell death	3.40%	0.03239
GO:0072330	monocarboxylic acid biosynthetic process	6.50%	0.04009
GO:0010200	response to chitin	4.30%	0.04212
**0 vs 24 h**			
GO:0006108	malate metabolic process	2.80%	8.1E-09
GO:0005992	trehalose biosynthetic process	3.00%	3.38E-06
GO:0005991	trehalose metabolic process	3.00%	1.66E-05
GO:0031408	oxylipin biosynthetic process	2.60%	2.17E-05
GO:0046351	disaccharide biosynthetic process	3.00%	0.000033
GO:0006833	water transport	3.30%	6.25E-05
GO:0042044	fluid transport	3.30%	6.25E-05
GO:0043067	regulation of programmed cell death	6.10%	6.27E-05
GO:0009719	response to endogenous stimulus	17.20%	8.23E-05
GO:0043648	dicarboxylic acid metabolic process	3.30%	0.00018
GO:0009312	oligosaccharide biosynthetic process	3.00%	0.00032
GO:0010941	regulation of cell death	6.10%	0.00035

GO:0015800	acidic amino acid transport	1.40%	0.00081
GO:0009963	positive regulation of flavonoid biosynthetic process	2.30%	0.00267
GO:0009725	response to hormone stimulus	15.20%	0.0029
GO:0055081	anion homeostasis	1.60%	0.0031
GO:1900378	positive regulation of secondary metabolite biosynthetic process	2.30%	0.00314
GO:0010566	regulation of ketone biosynthetic process	2.30%	0.00477
GO:1901700	response to oxygen-containing compound	19.80%	0.00588
GO:1900376	regulation of secondary metabolite biosynthetic process	2.30%	0.01131
GO:0006865	amino acid transport	4.70%	0.01159
GO:0010363	regulation of plant-type hypersensitive response	4.70%	0.01279
GO:0015804	neutral amino acid transport	2.30%	0.01477
GO:0009695	jasmonic acid biosynthetic process	3.00%	0.01869
GO:0010243	response to organic nitrogen	5.40%	0.02308
GO:0015976	carbon utilization	1.40%	0.02795
GO:0006612	protein targeting to membrane	4.70%	0.02982
GO:0080135	regulation of cellular response to stress	4.70%	0.03147
GO:0015802	basic amino acid transport	2.10%	0.03626
GO:0006633	fatty acid biosynthetic process	4.00%	0.04766
GO:2000762	regulation of phenylpropanoid metabolic process	2.30%	0.04901
**0 vs 48 h**			
GO:0009719	response to endogenous stimulus	19.30%	2.1E-11
GO:1901700	response to oxygen-containing compound	21.90%	5.58E-08
GO:0010243	response to organic nitrogen	6.70%	9.88E-08
GO:0009725	response to hormone stimulus	16.60%	1.13E-07
GO:0032879	regulation of localization	5.00%	2.65E-07
GO:0010033	response to organic substance	22.70%	1.67E-06
GO:0051049	regulation of transport	4.40%	2.03E-06
GO:0010200	response to chitin	5.00%	2.01E-05
GO:0042221	response to chemical stimulus	29.70%	2.29E-05
GO:0050896	response to stimulus	48.70%	5.95E-05
GO:0032870	cellular response to hormone stimulus	9.20%	7.56E-05
GO:0071495	cellular response to endogenous stimulus	9.20%	8.59E-05
GO:0043269	regulation of ion transport	3.60%	0.00011
GO:0009414	response to water deprivation	5.80%	0.00012
GO:0009415	response to water stimulus	5.80%	0.00026
GO:1901701	cellular response to oxygen-containing compound	9.90%	0.00049
GO:0016115	terpenoid catabolic process	0.90%	0.00091
GO:0002682	regulation of immune system process	5.10%	0.00103
GO:0051865	protein autoubiquitination	1.00%	0.00121
GO:0050776	regulation of immune response	5.00%	0.00246
GO:0044070	regulation of anion transport	1.40%	0.00317
GO:0010941	regulation of cell death	5.00%	0.00531
GO:0045487	gibberellin catabolic process	0.70%	0.00555
GO:0009628	response to abiotic stimulus	23.80%	0.00566
GO:0045088	regulation of innate immune response	4.80%	0.00668
GO:0043067	regulation of programmed cell death	4.60%	0.00804

GO:0009695	jasmonic acid biosynthetic process	2.70%	0.00875
GO:0010359	regulation of anion channel activity	1.20%	0.01205
GO:0033993	response to lipid	8.90%	0.01658
GO:0022898	regulation of transmembrane transporter activity	1.20%	0.01664
GO:0032409	regulation of transporter activity	1.20%	0.01664
GO:0032412	regulation of ion transmembrane transporter activity	1.20%	0.01664
GO:0010363	regulation of plant-type hypersensitive response	4.10%	0.01788
GO:0009611	response to wounding	4.40%	0.01896
GO:0009742	brassinosteroid-mediated signaling pathway	1.70%	0.01904
GO:0043401	steroid hormone-mediated signaling pathway	1.70%	0.01904
GO:0048545	response to steroid hormone stimulus	1.70%	0.01904
GO:0071367	cellular response to brassinosteroid stimulus	1.70%	0.01904
GO:0071383	cellular response to steroid hormone stimulus	1.70%	0.01904
GO:0046128	purine ribonucleoside metabolic process	7.20%	0.01965
GO:0042278	purine nucleoside metabolic process	7.20%	0.01992
GO:0009651	response to salt stress	7.70%	0.01992
GO:0009755	hormone-mediated signaling pathway	7.40%	0.02216
GO:0097305	response to alcohol	8.20%	0.02343
GO:0034762	regulation of transmembrane transport	1.20%	0.0249
GO:0034765	regulation of ion transmembrane transport	1.20%	0.0249
GO:0006970	response to osmotic stress	8.00%	0.02546
GO:0051707	response to other organism	12.80%	0.02558
GO:0071366	cellular response to indole-butyric acid stimulus	1.70%	0.02689
GO:0031347	regulation of defense response	6.50%	0.02727
GO:0080134	regulation of response to stress	6.70%	0.0305
GO:0060918	auxin transport	2.40%	0.03181
GO:0009926	auxin polar transport	2.20%	0.03452
GO:0009914	hormone transport	2.40%	0.03673
GO:0042388	gibberellic acid-mediated signaling pathway, G-alpha-dependent	1.00%	0.04099
GO:0080026	response to indole-butyric acid stimulus	1.70%	0.04156
GO:0046351	disaccharide biosynthetic process	1.90%	0.04281
GO:0070887	cellular response to chemical stimulus	12.50%	0.04337
GO:0005992	trehalose biosynthetic process	1.70%	0.0438
GO:0006612	protein targeting to membrane	4.10%	0.04636
GO:0080135	regulation of cellular response to stress	4.10%	0.04925
**GO terms for cold-repressed genes**			
**0 vs 12 h**			
GO:0006108	malate metabolic process	3.60%	0.00031
GO:0006833	water transport	4.80%	0.00213
GO:0042044	fluid transport	4.80%	0.00213
**0 vs 24 h**			
GO:2000377	regulation of reactive oxygen species metabolic process	5.40%	0.01863
**0 vs 48 h**			
GO:0006833	water transport	3.20%	0.01568
GO:0042044	fluid transport	3.20%	0.01568

The up- or down-regulated DEGs were annotated to the GO database using a hypergeometric test. The calculated p-value underwent a Bonferroni correction and the threshold of the corrected p-value was less than 0.05.

Table S9 Fold changes in transcript abundance of unigenes involved in terpene backbone biosynthesis under cold treatment.

Gene ID	12 h/0 h		24 h/0 h		48 h/0 h	
	log2 Ratio	Probability	log2 Ratio	Probability	log2 Ratio	Probability
**MVA**						
**AACT**						
Unigene26228_12h	-0.462114	0.411703166	-0.250732	0.323713837	-0.61072033	0.452453554
CL6110.Contig2_12h	-0.089976	0.232883073	-0.180004	0.288486318	0.300918274	0.336550179
CL6110.Contig1_12h	-0.079625	0.233927875	0.131684	0.275912103	-0.135789457	0.280737977
**HMGS**						
CL2821.Contig1_12h	0.4645687	0.414118925	0.344349	0.362725006	-0.346051196	0.354514939
CL2821.Contig3_12h	0.4168206	0.407517047	0.042301	0.23453216	0.548356506	0.433687006
CL2821.Contig4_12h	1.1786395	0.60248182	1.468668	0.642349945	2.211504105	0.708038375
CL2821.Contig5_12h	-0.474113	0.423057788	-0.566588	0.452539836	-0.124037349	0.272342052
**HMGR**						
CL5305.Contig1_12h	4.0796765	0.764753684	3.7040619	0.746596798	4.237217773	0.752822183
CL5305.Contig2_12h	4.042375	0.654637622	3.8754776	0.635932319	5.254919587	0.743623085
CL5305.Contig3_12h	1.2452971	0.625120581	1.8513934	0.695193306	3.447078611	0.753802608
CL5305.Contig4_12h	10.738374	0.987028422	9.8397287	0.965901328	9.703326841	0.954651201
CL5305.Contig5_12h	8.6450579	0.88158633	7.4093909	0.705877779	8.584962501	0.871924402
CL5305.Contig6_12h	-0.308426	0.278237988	1.3825571	0.59489117	3.334639147	0.746162773
CL5305.Contig7_12h	8.2667865	0.834090677	8.357552	0.855406246	9.944956027	0.96407048
CL5305.Contig8_12h	11.046215	0.990564995	10.688542	0.985792914	11.45943162	0.991270342
Unigene22810_12h	1.2734748	0.504056209	1.1129296	0.464326664	1.112929636	0.464326664
Unigene22812_12h	0.7621661	0.404349472	0.9350027	0.447835613	1.263563932	0.53482862
Unigene22813_12h	-0.471213	0.302329245	0.8163636	0.426341703	0.987704229	0.462149789
Unigene23291_12h	3.7539362	0.772396218	3.458775	0.761335677	3.58174937	0.756075033
Unigene23289_12h	-1.269001	0.636077183	-0.875981	0.536199466	0.236968653	0.321368828
MK						
Unigene20208_12h	-0.048081	0.215518075	-0.632903	0.463309007	-1.411528932	0.618941584
PMK						
CL10202.Contig3_12h	-0.803727	0.52068874	-1.099011	0.604499558	-1.791824509	0.675788217
CL10202.Contig1_12h	0.4133216	0.388510217	0.5000956	0.419153462	1.075916873	0.5870487
MVD						
Unigene30374_12h	0.5392311	0.4456081	-0.458458	0.412927448	-0.746006891	0.491412975
Unigene4484_12h	0.0612376	0.199489484	0.3016557	0.248589289	-0.519374159	0.268672383
Unigene35352_12h	-0.071385	0.209652811	-0.842606	0.412648361	-1.269587589	0.489071311
Unigene4483_12h	1.4720127	0.496152475	0.3016557	0.248589289	-0.519374159	0.268672383
Unigene37264_12h	0.7526317	0.351262193	0.0071421	0.197023343	-2.297084024	0.436514729
**MEP**						
**DXS**						
Unigene31120_12h	-0.329915	0.314189684	1.1437284	0.589034496	1.155144843	0.575014956
Unigene17149_12h	0.0143403	0.18377461	0.0904047	0.242077245	0.365264503	0.336667885
CL1050.Contig2_12h	-0.409777	0.400587632	-0.502475	0.430319743	-1.76524543	0.67936926

CL1050.Contig3_12h	-0.869437	0.519109099	-0.681062	0.464398976	-1.966052668	0.667461531
CL1050.Contig1_12h	3.670354	0.744022101	3.9526821	0.74856985	4.247618817	0.74917744
**DXR**						
Unigene23217_12h	-0.556718	0.454204223	-0.008384	0.20583057	-0.137531083	0.28097339
**MEP**						
Unigene25473_12h	-0.274502	0.317011755	0.3725142	0.361714181	-0.330124953	0.33556283
CL4811.Contig2_12h	-0.107384	0.213363517	-0.732239	0.359128802	-1.525338518	0.471873719
Unigene25476_12h	-0.957356	0.266868164	-6.058894	0.431300021	-1.785875195	0.321663786
CL4811.Contig1_12h	-0.485427	0.255560531	-1.392317	0.353322114	-2.299208018	0.398265701
CL4811.Contig3_12h	-0.081084	0.207676531	-7.81805	0.774717997	-0.755553597	0.355168556
Unigene25474_12h	-0.729427	0.386627915	-0.272133	0.27764078	-0.914706249	0.413304419
**CMK**						
Unigene18498_12h	-0.229986	0.317424977	-0.119456	0.26755336	-0.468042687	0.402964262
**MECS**						
Unigene21637_12h	-0.66756	0.489691562	-0.197069	0.306221571	-0.719129024	0.484934971
**HDR**						
Unigene21326_12h	-0.132121	0.261207437	-0.303054	0.356753087	-0.272704942	0.338317159
**HDS**						
Unigene22234_12h	0.0474747	0.219908732	-0.26088	0.337801512	-0.539195023	0.432649806
**Isomerization of IPP**						
**IDI**						
CL5444.Contig1_12h	-0.147328	0.267407149	-0.24781	0.330042599	0.161021828	0.289092359
CL5444.Contig2_12h	0.4403316	0.413509457	-0.38141	0.381444702	-0.369009402	0.369226849
Unigene1142_12h	-0.018547	0.197632058	-0.061401	0.240684584	0.498468298	0.41380571
CL1131.Contig2_12h	-0.302735	0.273219898	0.412459	0.318970736	2.653715192	0.719923948
**Prenyl transfer**						
**GGPPS**						
CL3490.Contig2_12h	0.1758516	0.283507605	-0.080914	0.251996657	-1.295285577	0.618404289
Unigene1243_12h	-0.796158	0.520073744	-0.061588	0.241785661	0.222564673	0.311136683
**FPPS**						
Unigene11224_12h	-0.420621	0.404168429	-0.947873	0.546360476	-1.501402538	0.640495696
**GPPS**						
Unigene41024_12h	-4.222392	0.261657974	2.1351596	0.359695309	3.047513461	0.488287525

Table S11 Sequence information and Genbank accession number of isolated genes

Gene ID	Annotation	Gene name	Full length (bp)	ORF (bp)	Amin acid length	Putative conserved domain	Accession No.
Unigene38249	CBF/DREB-like transcription factor 1 [*Citrus trifoliata*]	SaCBF1	798	645	215	AP superfamily	KX009411
CL5171.Contig2	AP2 domain class transcription factor [*Corylus mandshurica*]	SaCBF2	931	660	220	AP superfamily	KX009412
CL5171.Contig3	AP2 domain class transcription factor [*Corylus mandshurica*]	SaCBF3	939	660	220	AP superfamily	KX009413
CL5171.Contig5	CBF4 transcription factor [*Vitis amurensis*]	SaCBF4	1040	660	220	AP superfamily	KX009414
Unigene10585	AP2 domain class transcription factor [*Malus x domestica*]	SaERF017	1077	699	233	AP superfamily	KX229745
Unigene15122	Ethylene-responsive transcription factor [*Medicago truncatula*]	SaERF109-like	857	642	214	AP superfamily	KX229743
CL2204.Contig2	PREDICTED: ethylene-responsive transcription factor RAP2-4-like [*Vitis vinifera*]	SaRAP2.4	1933	1038	346	AP superfamily	KX009417
CL5042.Contig2	PREDICTED: zinc finger CCCH domain-containing protein 29-like	SaC3H29	2295	1857	619	ANK superfamily	KX229744
Unigene24270	ICE transcription factor 1 [*Eucalyptus globulus*]	SaICE1	1946	1629	543	bHLH family	KX009419

ORF, open reading frame

Table S12 The primers for RACE and isolation of full lengths of cold-induced transcription factor genes

Gene ID	Primer name	Primer sequence (5' to 3')
**3' UTR**		
Unigene38249	CBF1outer	CCTCAGAGTGAAGAAGAGAAGGTG
	CBF1inner	GCAGAGTATATGGACGAGGAAGC
CL5171.Contig2	CBF2outer	CTCATCAGCTTCGTCGGCTTC
	CBF2inner	GTCCAACAACCCGAAGAAG
CL5171.Contig3	CBF3outer	GCACTTGGAAACCCCAGTAAC
	CBF3inner	GCCCCAACTTTTCTGGTCCTC
CL5171.Contig5	CBF4outer	GCGAGCCCAGCAAGCAGGCGAGGAT
	CBF4inner	TCAATTTTGAGGACTCGGCGTGGCG
Unigene10585	AP2outer	AGTAGTAGTGGGAGGGGTGCGTA
	AP2inner	GATATGGTTGGGCTCCTACGACT
Unigene15122	ERFouter	AGGAAGAAGACGGCCAAGAAG
	ERFinner	GCAACACCGAGGATTTTAACG
CL2204.Contig2	RAP2.4outer	GAACTACAAGCCTCTCCACTCCTC
	RAP2.4inner	CGTTCTGCTTCTCAGATCCTACC
CL5042.Contig2	ZFouter	GGTGCTGCTAATTCTGCTGCT
	ZFinner	ACTGCTGAGGAGCCTGATGTC
Unigene24270	ICE1outer	CTGAGGTCCGTCGTCCCAAAGAT
	ICE1inner	CCACCCTTTGACACCAACACTAC
**5' UTR**		
Unigene38249	CBF1GSP1	TCCCTTACCTCGCACACCCACTTAT
	CBF1GSP2	CAGGTGAACCTCTTCGTCGGAAAAC
CL5171.Contig2	CBF2GSP1	ACCGCTTCTTCGGGTTGTTGGAC
	CBF2GSP2	CATTCGAAGCCGACGAAGCTGAT
CL5171.Contig3	CBF3GSP1	GAGGACCAGAAAAGTTGGGGCAAA
	CBF3GSP2	GGCGGGAGTGGGAGAAAAACTAGTAA
CL5171.Contig5	CBF4GSP1	TCTGTACACAGGGTGCCGAGTCTCT
	CBF4GSP2	CTGTACACAGGGTGCCGAGTCTCTC
Unigene10585	AP2GSP1	GAGTTGGATCTGGGAAGGGGTGAG
	AP2GSP2	AGAAGAGGGCGGCGTCGAAAGCGCG
Unigene15122	ERFGSP1	AATCCTCGGTGTTGCTGCTTTCC
	ERFGSP2	TTGGCCGTCTTCTTCCTCTTTCC
CL2204.Contig2	RAP2.4GSP1	CTGCCTCACTCCCCTGTAGAGCTTC
	RAP2.4GSP2	CTGAAGGATTTGAGATGGGGTGAGG
CL5042.Contig2	ZFGSP1	TTTCAAGCAACGATTCGGAACCA
	ZFGSP2	CAGCGAGATCATCGGAAGCAGAG
Unigene24270	ICE1GSP1	GAACTGCAACTGAGATGGGTCGAGA
	ICE1GSP2	CAACTGAGATGGGTCGAGATTGTGG
**ORF**		
Unigene38249	CBF1_F	ATGGAAAATTGGTCTGCACAT
	CBF1_R	CTACTGGAAACTCCACAACGA
CL5171.Contig2	CBF2_F	ATGGCGGCGGGAGCAACCCGA
	CBF2_R	TTATATGGAGTAGCTCCATAA
CL5171.Contig3	CBF3_F	ATGGATGTTTGCCCCAACTTT
	CBF3_R	TTATATGGAATAGCTCCACAA
CL5171.Contig5	CBF4_F	ATGGATGTTTTCTCCAACTTTT
	CBF4_R	TTATATGGAGTAGCTCCACAAG
Unigene10585	AP2_F	ATGGCGAAACAACAGCCGCGAGGC
	AP2_R	ATCAAATCGAAAAATCCCAGAGGAA
Unigene15122	ERF_F	ATGGAGAGTCCCTTCCAGAGACC
	ERF_R	TTAGACATAGCAAAACAAAACT
CL2204.Contig2	RAP2.4_F	CCACTCAAAAGGAGAGTTTCATG
	RAP2.4_R	CTAAATCGCCGCCCAATCAATCT
CL5042.Contig2	ZF_F	ATGTGCCAGGGATCCAAGACTGA
	ZF_R	TTAAGCCACCATCTGCTCTTGTT
Unigene24270	ICE1_F	ATGCTGTCTAGGGTGAACGGCGT
	ICE1_R	TTACATCACGCCATGGAAGCCGG

For the completion of 3' and 5'-UTR of each gene, two sense primers were needed for 3' and 5'-RACE PCR.

Table S13 Primers used for qRT-PCR

Gene ID	Primer name	Primer sequence (5' to 3')
Unigene38249	SaCBF1_F	GGAATGGTGAGAGGAGTGTAAA
	SaCBF1_R	CATCGCTTCCTCGTCCATATAC
CL5171.Contig2	SaCBF2_F	ATCAGCTTCGTCGGCTTC
	SaCBF2_R	CGAGTCTCTCGGAACTTCTTC
CL5171.Contig3	SaCBF3_F	AGGCAGCAGAGGCATTTC
	SaCBF3_R	CGTCCATATACGCCCAGTTC
CL5171.Contig5	SaCBF4_F	GCATTTCGCCCGCTAGAA
	SaCBF4_R	CGCCTCCTCGTCCAGATA
Unigene10585	SaERF017_F	GGAGTGAGGAAGCGAAAGT
	SaERF017_R	CGTAGGAGCCCAACCATATC
Unigene15122	SaERF109-like_F	TAAACACGGCCGAGGAATG
	SaERF109-like_R	CTCAAACTCTTGTCCCTCAAAGA
CL2204.Contig2	SaRAP2.4_F	AATAGGTAGCGGAGATCAGACT
	SaRAP2.4_R	GAAATTGAGCCTCGCGAAATC
CL5042.Contig2	SaC3H29_F	TGGGCATTTCGGTTCAGAT
	SaC3H29_R	CCACCATCTGCTCTTGTTCTAT
Unigene24270	SaICE1_F	GATGGAGATCAGAAGGGAAAGAA
	SaICE1_R	ACGGACCTCAGCATGTAAAG
CL3743.contig8	SaTPS1_F	GGCTCATGGACCAATCTTCT
	SaTPS1_R	CCGTCTCCGTGCTGATAAAT
Unigene 61780	SaTPS2_F	AGAAGGAGAAGACATACTTGATGAG
	SaTPS2_R	CCCAACAACTCTAGCTTGAAGA
CL16081	SaTPS3_F	CGAGGGTGCACTGGATAAA
	SaTPS3_R	CGTCCGAAGAGGTTGCTAAA
CL425.Contig12	SaTPS4_F	AGGCTTGTCGGAGAAAGATG
	SaTPS4_R	GGATCTAGGGAGTGAGGTTCTA
CL975.Contig15	SaTPS5_F	AGGCTTAAGTGGGCCTAGTA
	SaTPS5_R	GTGCCGTAGATGTCTCGTTATG
Unigene 64433	SaTPS6_F	GTCAAACAGGCCAGTCTCATT
	SaTPS6_R	TGTCCCACCTGTCAACAATATC
CL5305. contig4	SaHMGR_F	GTCTGTGACCATGCCATCAA
	SaHMGR_R	CACCTGCCACAACAACATTTC
CL737.Contig6	SaCYP76F38_F	CGTCGGATGACGGGTTATTT
	SaCYP76F38_R	CACCATCACTTTGTTGCTTTCT
	SaActin_F	GTCACACGGTGCCAATCTAT
	SaActin_R	TACCCTCTCTCAGTCAGAATCTT

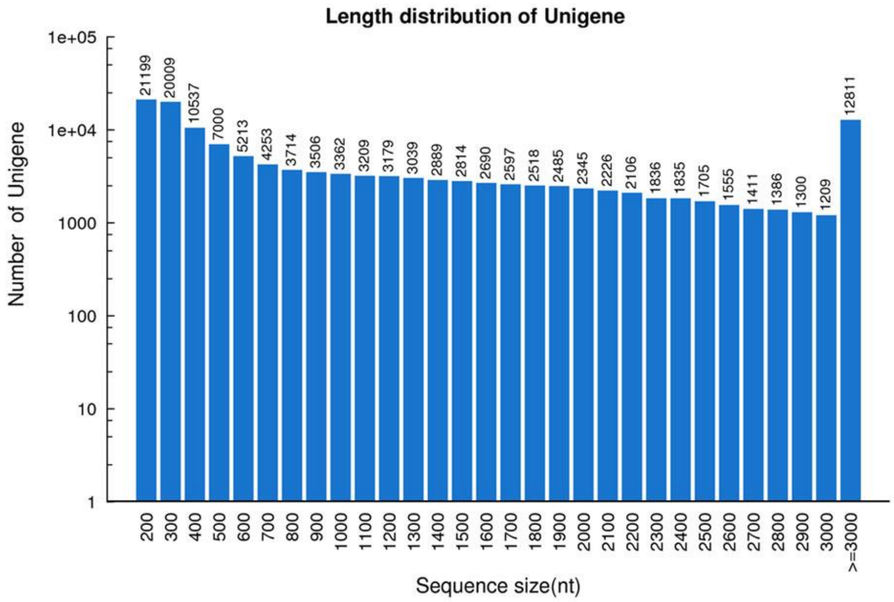

Figure S1. Length distribution of unigenes.

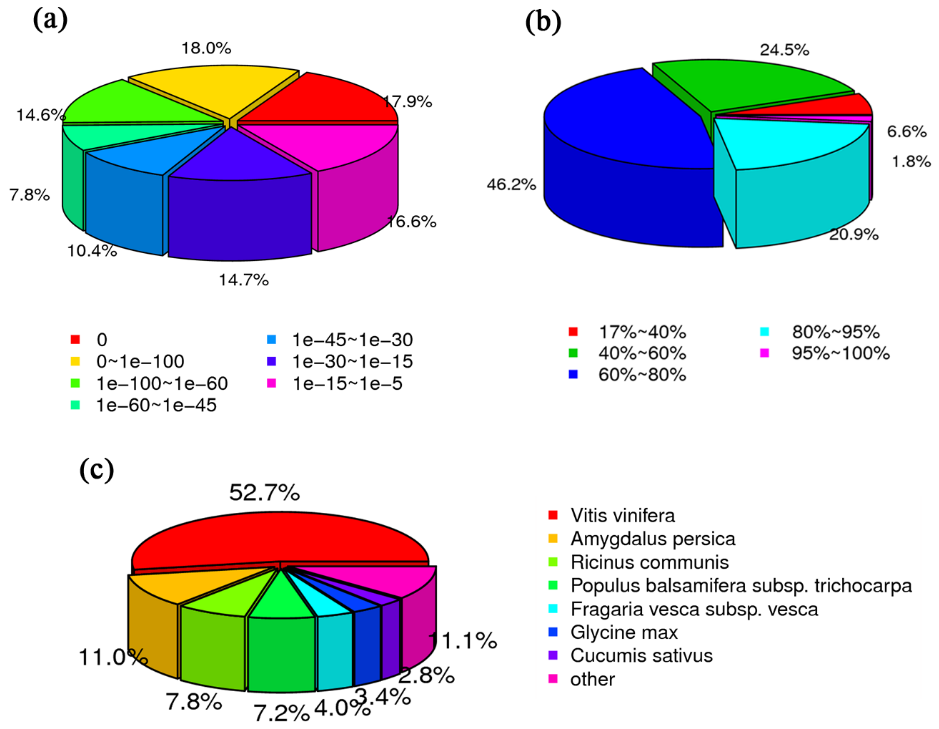

Figure S2. Characteristics of homology search of query sequences aligned by BLASTx to the nr database with a cut-off E-value of $1.0E^{-5}$. (a) E-value distribution. (b) Similarity distribution. (c) Species distribution of the first Blast hits for each sequence.

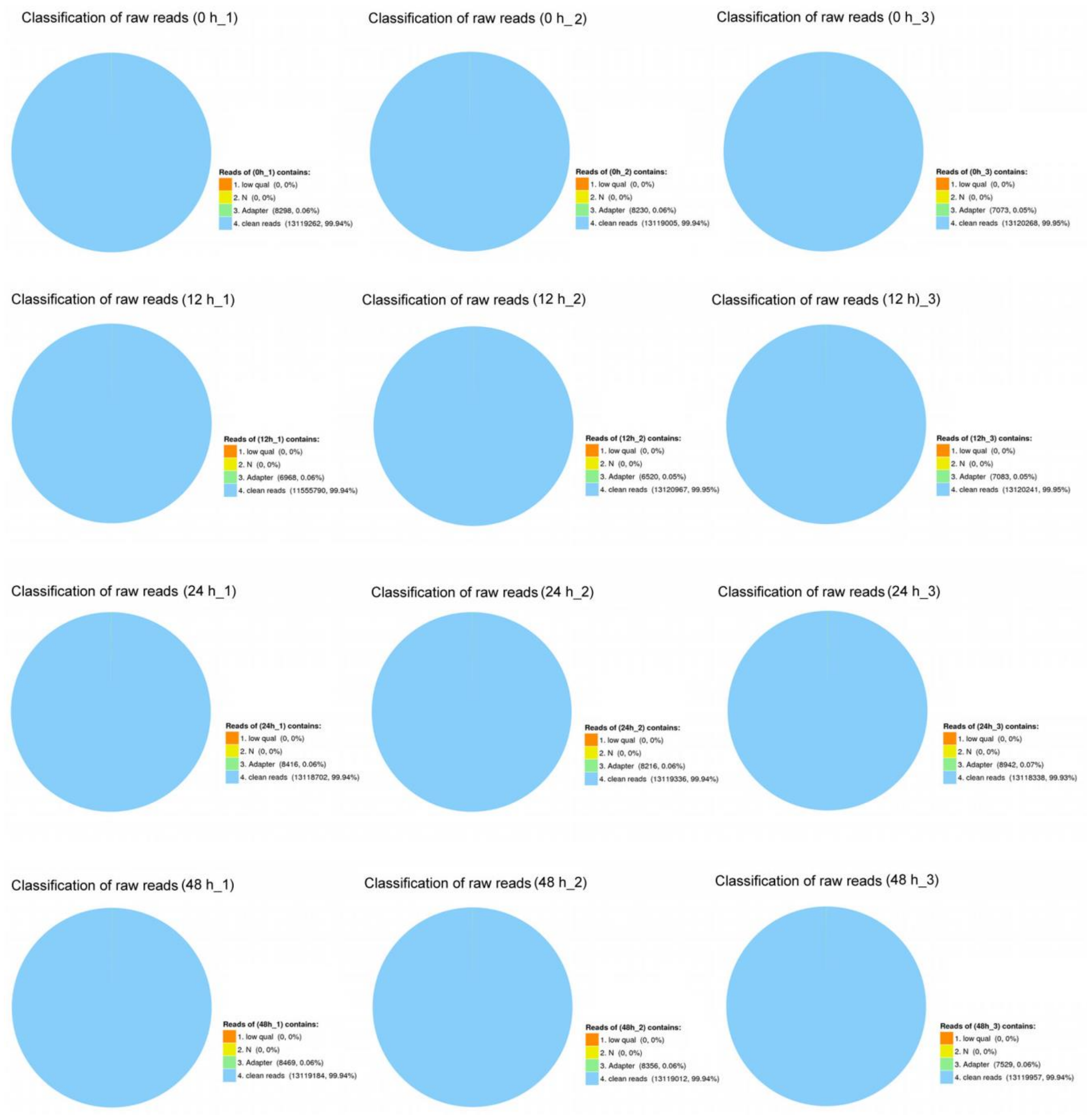

Figure S3. Pie charts of the component of raw data in all samples. The values in the legends indicate the number of reads and the ratio to raw reads.

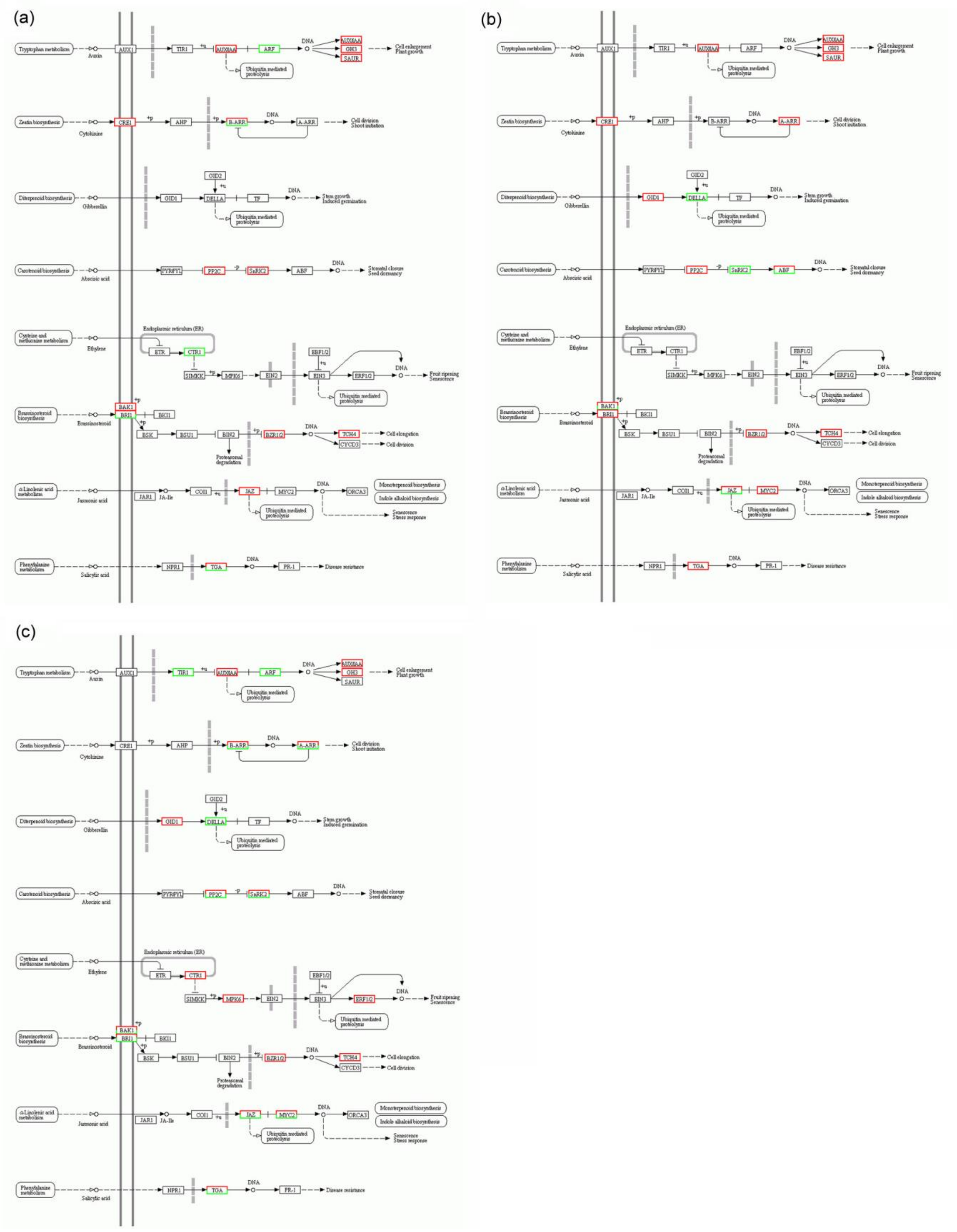

Figure S4. Differential expression of genes involved in hormone signal transduction in response to cold stress. (a) 0 h vs 12 h; (b) 0 h vs 24 h; (c) 0 h vs 48 h.

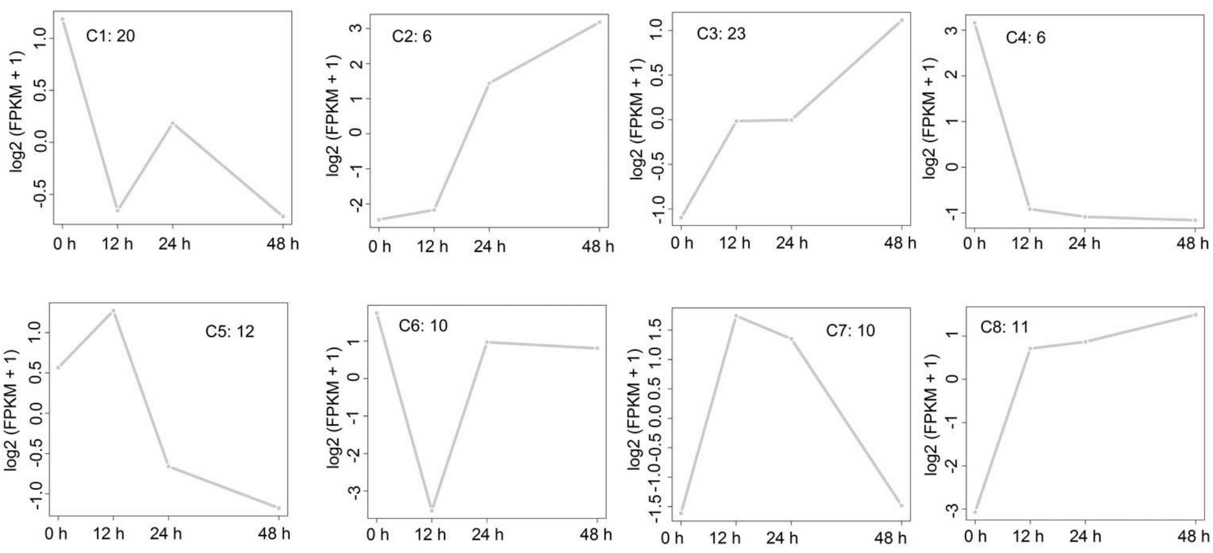

Figure S5. Expression models of DEGs related to photosynthesis under cold stress.

(a)

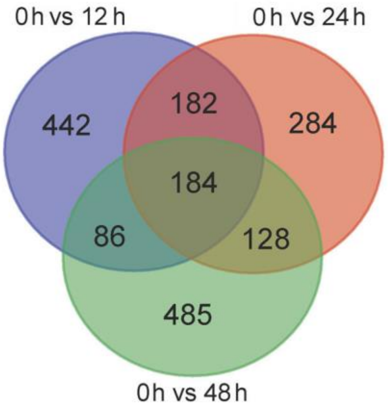
0h vs 12h
0h vs 24h
442
182
284
184
86
128
485
0h vs 48h

(b)

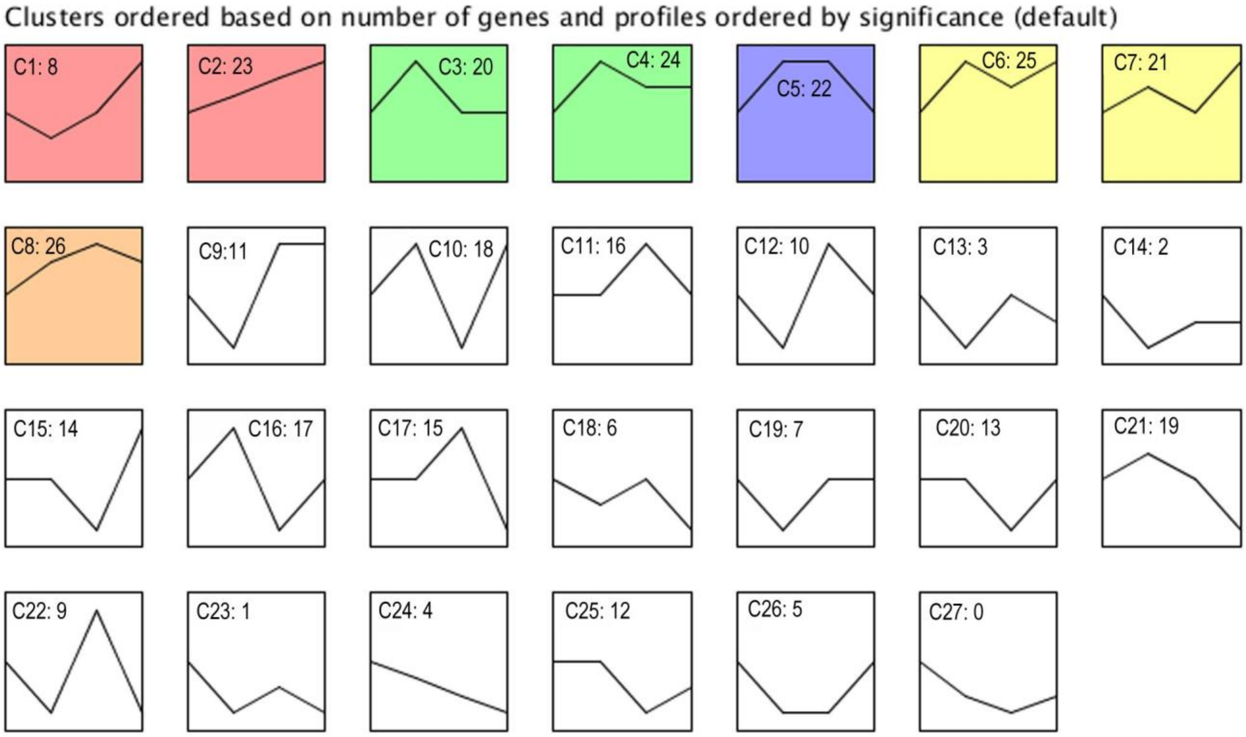
Clusters ordered based on number of genes and profiles ordered by significance (default)
C1: 8
C2: 23
C3: 20
C4: 24
C5: 22
C6: 25
C7: 21
C8: 26
C9:11
C10: 18
C11: 16
C12: 10
C13: 3
C14: 2
C15: 14
C16: 17
C17: 15
C18: 6
C19: 7
C20: 13
C21: 19
C22: 9
C23: 1
C24: 4
C25: 12
C26: 5
C27: 0

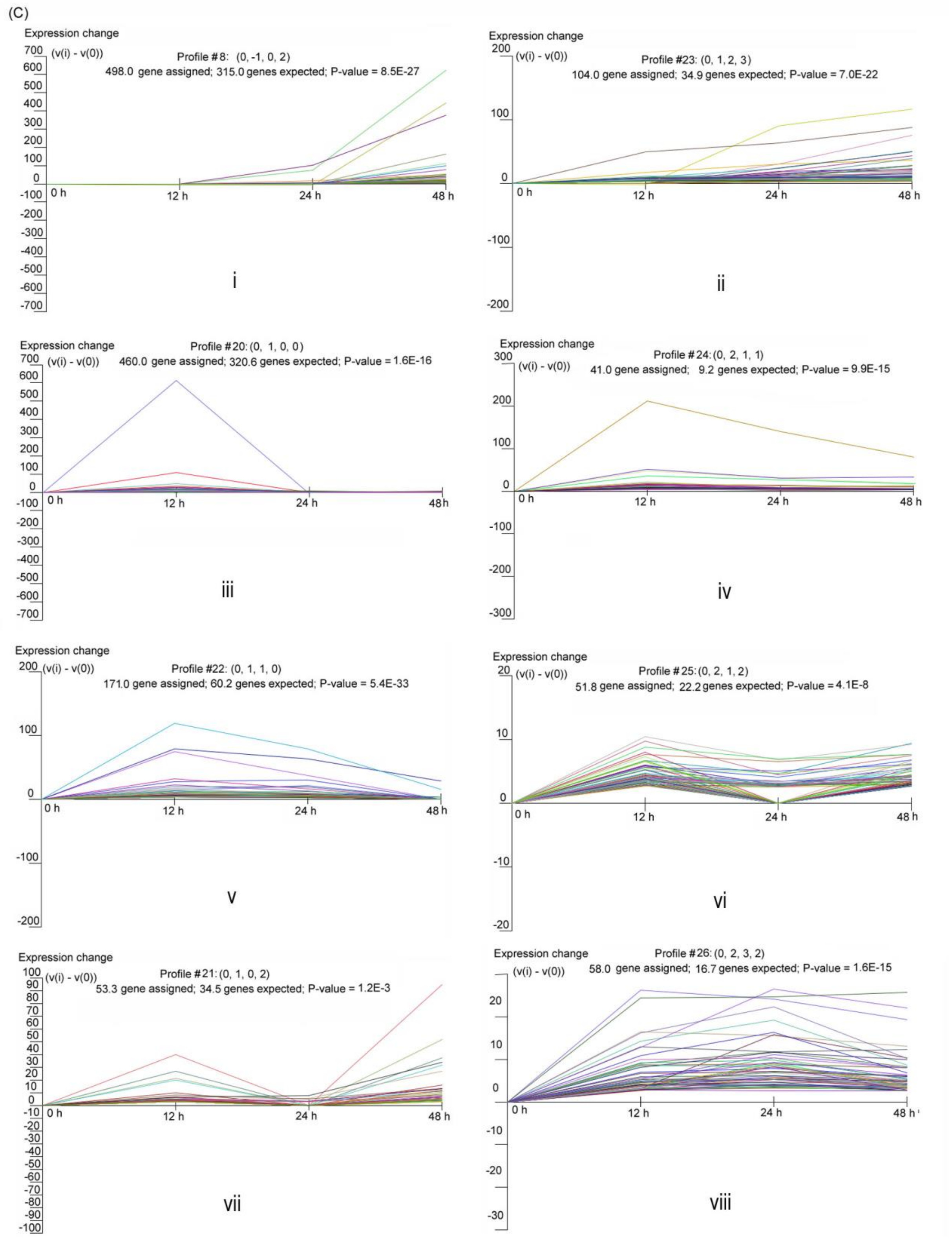

Figure S6. Novel genes involved in the cold stress response of *S. album*. (a) Venn diagram of number of novel genes after exposure to 4°C. (b) Expression models of these genes consisting of 27 clusters. Blots in colors represent significant clusters (P-value ≤ 0.05). (c) Expression profiles of correspondingly significant clusters in (b).

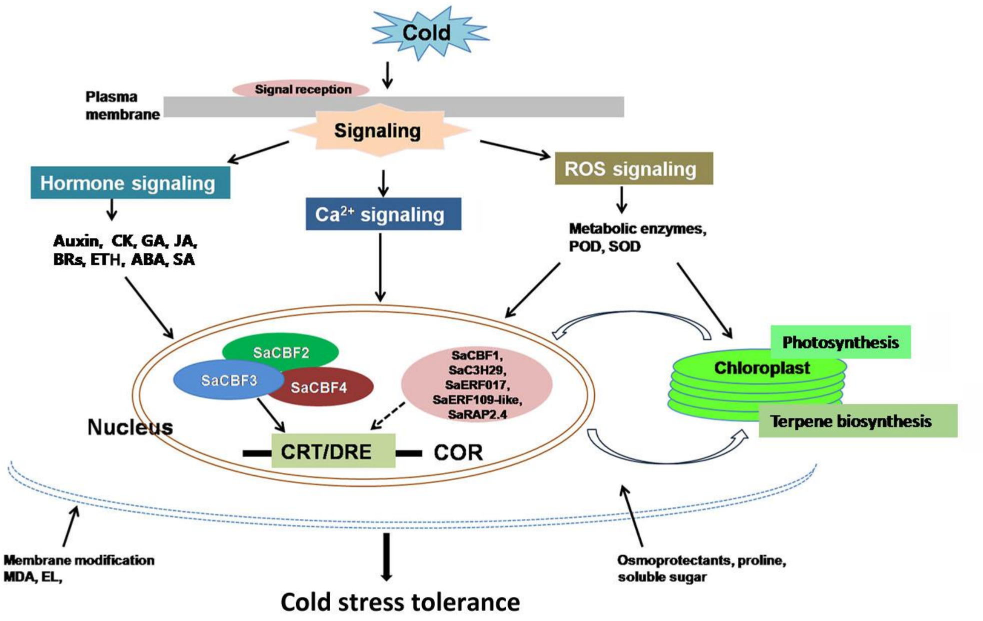

Figure S7. A model for sandalwood cold stress response networks. After signal reception, stress-activated Ca^{2+} signaling, ROS signaling, and hormone signaling modulate the expression of stress-responsive genes, inducing the stress response. Our study shows that *SaCBF2 to 4* are critical contributors to the cold regulatory mechanism. Dotted arrow represents transcription factors that might be involved in the regulation of *COR* genes during cold stress. BR, brassinosteroid; CK, cytokinin; EL, electrolyte leakage; ETH, ethylene; GA, gibberellin; JA, jasmonic acid; MDA, malondialdehyde; POD, peroxidase; SA, salicylic acid; SOD, superoxide dismutase.
